# Air Pollution Forecasts: An Overview

**DOI:** 10.3390/ijerph15040780

**Published:** 2018-04-17

**Authors:** Lu Bai, Jianzhou Wang, Xuejiao Ma, Haiyan Lu

**Affiliations:** 1 School of Statistics, Dongbei University of Finance and Economics, Dalian 116025, China; baildctg@hotmail.com (L.B.); xuejiaomadufe@163.com (X.M.); 2 Faculty of Engineering and Information Technology, University of Technology, Sydney, NSW 2007, Australia; haiyan.lu@uts.edu.au

**Keywords:** air pollution forecast, forecasting models, statistical methods, artificial intelligence methods, numerical forecast methods, hybrid models

## Abstract

Air pollution is defined as a phenomenon harmful to the ecological system and the normal conditions of human existence and development when some substances in the atmosphere exceed a certain concentration. In the face of increasingly serious environmental pollution problems, scholars have conducted a significant quantity of related research, and in those studies, the forecasting of air pollution has been of paramount importance. As a precaution, the air pollution forecast is the basis for taking effective pollution control measures, and accurate forecasting of air pollution has become an important task. Extensive research indicates that the methods of air pollution forecasting can be broadly divided into three classical categories: statistical forecasting methods, artificial intelligence methods, and numerical forecasting methods. More recently, some hybrid models have been proposed, which can improve the forecast accuracy. To provide a clear perspective on air pollution forecasting, this study reviews the theory and application of those forecasting models. In addition, based on a comparison of different forecasting methods, the advantages and disadvantages of some methods of forecasting are also provided. This study aims to provide an overview of air pollution forecasting methods for easy access and reference by researchers, which will be helpful in further studies.

## 1. Introduction

Air is a basic requirement for the survival and development of all lives on Earth. It affects health and influences the development of the economy. Today, due to the development of industrialization, the increase in the number of private cars, and the burning of fossil fuels, air quality is decreasing, with increasingly serious air pollution. There are many pollutants in the atmosphere, such as SO_2_, NO_2_, CO_2_, NO, CO, NO_x_, PM_2.5_, and PM_10_. Internationally, a large number of scholars have conducted research on air pollution and air quality forecasts, concentrating on the forecasting of contaminants. 

Air pollution affects the life of a society, and even endangers the survival of mankind. During the Industrial Revolution, there was a dramatic increase in coal use by factories and households, and the smog caused significant morbidity and mortality, particularly when combined with stagnant atmospheric conditions. During the Great London Smog of 1952, heavy pollution for 5 days caused at least 4000 deaths [1,2]. This episode highlighted the relationship between air pollution and human health, yet air pollution continues to be a growing problem in cities and households around the world. 

Air pollution is made up of a mixture of gases and particles in harmful amounts that are released into the atmosphere due to either natural or human activities [3]. The sources of pollutants can be divided into two categories:

(1) Natural sources

Natural pollution sources are natural phenomena that discharge harmful substances or have harmful effects on the environment. Natural phenomena, such as volcanic eruptions and forest fires, will result in air pollutants, including SO_2_, CO_2_, NO_2_, CO, and sulfate.

(2) Anthropogenic (man-made) sources

Man-made sources such as the burning of fuels, discharges from industrial production processes, and transportation emissions are the main sources of air pollution. There are many kinds of pollutants emitted by man-made pollution sources, including hydrogen, oxygen, nitrogen, sulfur, metal compounds, and particulate matter.

With the increasing world population and the developing world economy, the demand for energy in the world has increased dramatically. The large-scale use of fossil energy globally has also led to a series of environmental problems that have received much attention due to their detrimental effects on human health and the environment [3,4,5]. Air pollution is a fundamental problem in many parts of the world, with two important concerns: the impact on human health, such as cardiovascular diseases, and the impact on the environment, such as acid rain, climate change, and global warming [6]. These environmental impacts are described below.

(1) Climate change

Some chemicals released into the atmosphere by human activities, such as CO_2_, CH_4_, N_2_O, and chlorofluorocarbons (CFCs, exemplified byFreon-12), cause a greenhouse effect [7,8]. The burning of fossil fuels and other human activities increase the concentration of greenhouse gases, leading to global warming. This also leads to a rise in sea level, more extreme weather, and melting glaciers and ice caps. More alterations to the environment are inevitable as temperatures continue to climb [7].

The studies have indicated that the rate of sea level increase was the fastest in the twentieth century, and data have proven this point of view. The sea level has risen 14 cm in the twentieth century. A study shows that the sea level will rise by 28 cm and is expected to reach a total of 131 cm in 2100 [3,7,9], while average global temperature will increase by 3.6 °F to 8.1 °F (2 °C to 4.5 °C) [7].

(2) Ozone Hole

The ozone layer is a relatively high level concentration of ozone in the stratosphere, and its main function is to absorb ultraviolet radiation. It has many useful functions for Earth, and the most important of those functions is to protect human beings, animals, and plants from short wave ultraviolet radiation [10]. It also protects against the heating effect, as ozone absorbs the Sun’s ultraviolet rays and converts it to heat energy that heats the atmosphere [11].

Freon, a halohydrocarbon, and N_2_O can produce the greenhouse effect and can also react with stratospheric ozone, resulting in the depletion of the ozone layer and creation of holes in the ozone layer [10,12].

The decline of the stratospheric ozone level from anthropogenic source is internationally recognized as one of the Earth’s most important environmental issues [13]. The ozone hole is affecting human health and the environment negatively and can cause severe diseases, such as skin cancer, eye damage, and genetic mutations [10,12]. Research results show that if stratospheric ozone concentrations decreased by 1%, the amount of ultraviolet radiation will be increased by 2%, and the cataract rate will increase 0.2–0.6%. Moreover, the depletion of the ozone layer seriously harms the human body, crops, and forests, even destroying natural biosphere generation and the marine ecological balance [12].

In recent years, scientists discovered that the phenomenon of ozone reduction occurs in both the Antarctic and Arctic [11]. In the spring of 2011, ozone column loss had reached 40%. According to the observations of Chinese atmospheric physics and meteorology over the Qinghai-Tibetan Plateau, the ozone layer is being reduced at a rate of 2.7% per 10 years.

(3) Particulate matter pollution

Atmospheric particulate matter consists of solid or liquid granular substances in the atmosphere. Thick smog along with particulate matter (PM) occurs and covers most cities of world frequently [4]. According to medical research, PM causes different degrees of harm to human respiratory, cardiovascular, and central nervous, and immune systems and to genes [14,15].

China, as the largest developing country, has attracted great attention from all over the world for its rapid economic development and its air pollution. In 2015, China’s air pollution situation was very serious with most cities’ air quality exceeding the China National Standard. Moreover, some cities in China have been selected as the 10 most polluted cities in the world [16]. In recent years in China, high concentrations of particulate matter have received increasing attention [17].

Generally, air pollutants do not just harm the local or regional environment. They can also cause damage on a global scale. Certain man-made chemicals have damaged the planet’s protective ozone layer, allowing more harmful solar radiation to strike the Earth’s surface. Although the use of these chemicals is being phased out, their destructive effects will linger for many more decades. 

Control of air pollution and improving air quality are presently concern of scientists globally [18]. As one of the important results of urban air pollution control, urban air pollution forecasting has established an urban air pollution alarm system, effectively reducing the cost of air pollution control. The establishment of a reasonable and accurate forecasting model is the basis for forecasting urban air pollution. Forecasting is a requisite part of in the science of big data and can be used to infer the future development of an object relative to previous information. So “pollution forecasting” can be understood as estimation of pollutant concentration at specified future date. 

Since the 1960s, with the development of air pollution control and research, it has become urgent for people to understand the influence of air pollution and the trends of pollution. Therefore, forecasting air pollution began. Forecasting pollution using different patterns of performance can be divided into three types: potential forecasts, statistical models, and numerical models. For different elements, it is divided into pollution potential forecasting and concentration forecasting [19]. Statistical methods and numerical modelling methods result in concentration forecasts. A potential forecast is mainly based on the meteorological conditions for atmospheric dilution and diffusion capacity. When the weather conditions are expected to be in line with the standards for possible serious pollution, a warning will be issued. A concentration forecast will forecast the concentration of pollutants in a certain area directly, and the forecast results are quantitative. These air pollutions forecasting models can be divided into parametric and nonparametric models, or deterministic and nondeterministic models. It is easy to distinguish the parametric models from nonparametric models, and deterministic models from nondeterministic models, but it is difficult to differentiate the parametric models from deterministic models. The most significant difference between parametric models and deterministic models is that for a deterministic model, the output can be determined, as long as inputs are fixed, regardless of the number of trials; while the parametric model is to determine the parameters of equations in the known model, and its output is uncertain. For example, the diffusion models in this paper belong to the deterministic model, and they are based on physical equations, driven by the chemistry and the transport of pollutants, requiring many accurate input data [20]; models based on large amounts of historical data, such as regression, principal component analysis, etc., are usually parametric models.

The most popular statistical method uses artificial intelligence (AI) models. The accuracy of neural network (NN) forecasting models is higher than that of other statistical models [21,22,23], but they should be improved. Therefore, some scholars have been improving the forecast accuracy by other methods. Grivas et al. developed an artificial neural network (ANN) that combined meteorological and time-scale input variables [22]. Elangasinghe et al. built an ANN air pollution forecast tool based on meteorological parameters and the emission pattern of sources [23]. The improved ANN models were found to be more effective based on the same input parameters [24,25,26].

A commonly used numerical model is the Community Multi-scale Air Quality (CMAQ) modeling system. Since the 1970s, three generations of CMAQ models have been developed. Lou et al. used the CMAQ modeling system to analyze and evaluate air pollutant ozone concentrations in China and proposed that this method could be applied to other oxides of nitrogen [27].

**Figure 1:**
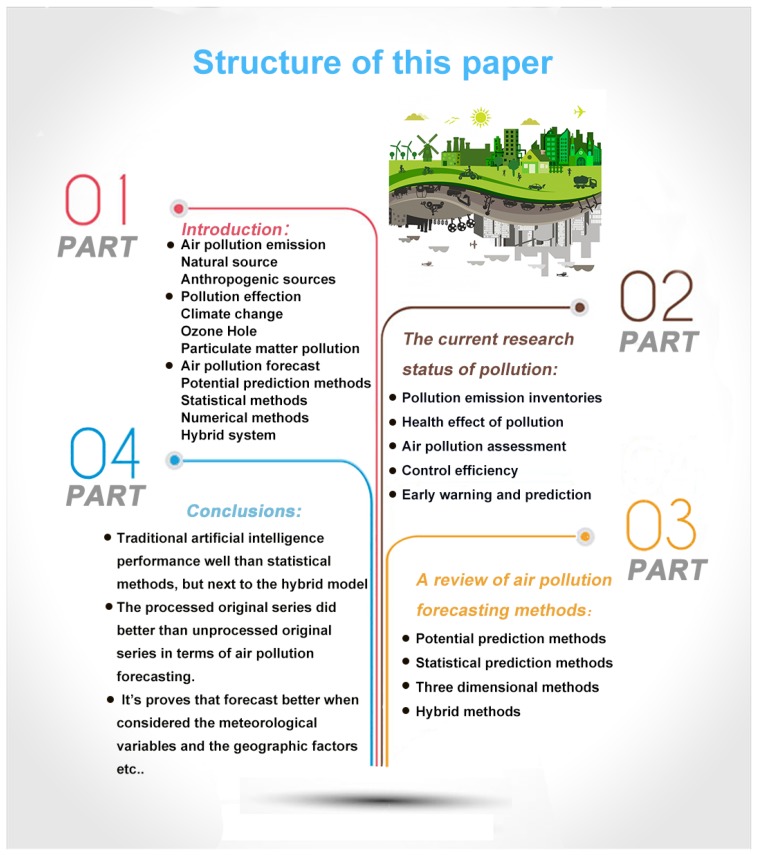
The construction of this paper.

Up to now, a large number methodologies and approaches have been proposed for air pollution forecasting but no comparison of these methods in the accuracy of forecast have been made. In the present paper, we have discussed various approaches and given statistical analysis to find out an accurate method. Figure 1 shows the plan of the study.

## 2. The Current Status of Pollution Research

Air pollution is regarded as an unavoidable reality. Over the past few years, much news about environmental pollution accidents have been reported, especially air pollution events. If the environmental problems are ignored in process of social progress, the ecological environment of the earth will gradually deteriorate, so the Earth is always in danger and every day will be “2012”. It is well-known that, compared with land pollution and water pollution, the consequences of air pollution are more serious. Scholars have conducted a series of studies on air pollution, from pollution sources to pollution management and pollution forecasts, including the problem of emissions inventories, pollution assessments, and pollution alarms. These topics lay the foundation for the research into air pollution covered in the following sections. 

### 2.1. The Current Status of Pollution Emission Inventory Research

In the words of Seika, the emission inventory (EI) is a comprehensive list of various types of air pollutants emitted by various sources of pollution in a given area within a given time interval [28]. EIs provide a description of the polluting activities that occur across a specific geographic domain and are widely used as input for air quality modeling for the assessment of compliance with environmental legislation [29]. Air pollution control requires complex environmental management, in which clear EIs are the basis for other research. 

United States Environmental Protection Agency (EPA) developed an emission inventory improvement program (EIIP) in 1993. This program promoted the development and usage of collection, storage, reporting, sharing and other standardization process of data. The EIIP documents were designed to provide standardized approaches for emission estimation, the emission estimates formula is as follows [30]:

(1) Emissions=Activity Level×Emission Factor×(1−Level of Control)

For point sources, activity levels represent the operating rate of the facility, estimated at the facility level. For area sources, replacing emissions with some other variable, such as population count in a region, is used as an activity level. The correlates between surrogate activity factor and the emission rate for the source determine the quality of the estimates. The emission factor is the value of the amount of pollutants released into the atmosphere per unit activity associated with the release of the contaminant. And the level of control is equal to the amount controlled, one minus the level of control is represents the amount emitted after control [30].

### 2.2. The Health Effect of Pollution

Exposure to air pollution has been clearly associated with a range of adverse health effects. A report from the OECD indicated that outdoor air pollution could cost the world $2.6 trillion a year, by 2060, which includes the cost of sick days, medical bills and reduced agricultural output. Moreover, welfare costs associated with premature death by 2060 will rise to as much as $25 trillion [31]. Lafuente et al. performed a systematic review to assess the effects of air pollutants on sperm quality [32]. They set up four semen quality parameters, including DNA fragmentation, sperm count, sperm motility, and sperm morphology. Most studies concluded that air pollution impacted at least one of the four semen quality parameters included in the review. 

Wei et al. studied the effects of ambient NO_2_, SO_2_, and PM_10_ on childhood eczema in Shanghai, China. They selected 3358 preschool children for their 6-year research program. This study indicated that gestational and lifetime exposures to NO_2_ were risk factors for atopic eczema in childhood; moreover, exposure to SO_2_, and PM_10_ may enhance the effect of NO_2_ exposure on childhood eczema [33].

Beelen et al. developed a multi-center cohort study for Europe. The results indicated that the risk of natural mortality was significantly increased when exposed to PM_2.5_ for a long time [34]. The study showed that there is a positive correlation between PM_2.5_ and heart disease mortality. In addition, as the PM_2.5_ concentration increased, the mortality rate of patients with heart disease increased.

Various studies have testified that air pollution is harmful to human and other kind of creatures, and lead to varies diseases and loss, such as respiratory disease, cardiovascular disease, Death of animals and plants and economic losses.

### 2.3. Air Pollution Assessment

In recent years, air pollution accidents have occurred frequently, which have damaged the economy and human life. To assess the extent of the damage, air pollution control must be evaluated in order to have a quantitative understanding of pollution.

The assessment of air pollution is identify and measure the degree and scope of damage caused by environmental pollution cover the economic, legal, technical and other means reasonably [35,36,37].

Two of the more mature assessment methods will be described. The market value method is a type of cost benefit analysis method. It uses the change of product yield and profit caused by the environmental quality change to measure the economic loss related to the environmental quality change.

Environmental pollution and damage caused by air pollution can be prevented, restored, or replaced by the existing environmental functions. Therefore, the cost of preventing, restoring, or replacing the original functional protection facilities can be used to estimate the loss caused by pollution or damage to the environment. This method is called the engineering cost method.

**Figure 2:**
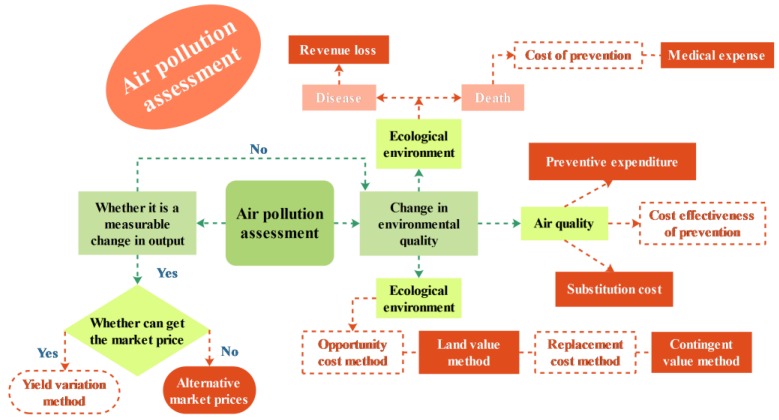
The flowchart of the assessment methods.

The main equation and the meaning of the variables in those methods are given in Table 1, and the flowchart of the assessment methods is given in Figure 2. 

### 2.4. Study of Air Pollution Control Efficienc

In order to solve increasingly serious environmental pollution problems, many countries have introduced policies to control pollution. In addition, the United Nations has organized international conferences, appealing to all countries to jointly manage global pollution. So, are these pollution control methods effective? The efficiency of environmental pollution control is the input and output efficiency in the process of environmental pollution control, reflecting the input of environmental pollution control and its pollution control effect.

Larsson et al. calculated air pollution control efficiency of the different enterprises in Norway [38]. They examined the effect of both technical efficiency and environmental efficiency. The governance efficiency of SO_2_ in each province of China was calculated by Shi et al. using the data envelopment analysis (DEA) method [39]. Wang et al. used a super efficiency DEA model to analyze the atmospheric pollution governance efficiency in various provinces of China from 2004 to 2009 [40]. Xie et al. studied Beijing and built an odd-and-even license plate model by a probabilistic modeling method and the analysis of means to quantify the pollution caused by vehicle exhaust emissions and the actual effect of the license plate limitation rule [41]. Fan et al. indicated that the rate of industrial waste gas governance is low, and there are significant differences in the governance efficiency of different pollutants [42]. Moreover, the Fan et al. research on China’s industrial air pollution control showed that, in different sectors, the air pollution treatment efficiency and its contributions from efficiency change and technology change differ significantly, and the contribution of technology advancement to the efficiency of industrial air pollution treatment are weak [43].

**Remark** **1.** *There is much air pollution control efficiency research for different industries and different pollutants, and the main result of that research is to take pollution management related costs as input variables and pollutant emission reductions as output variables and use a DEA model to calculate pollution abatement efficiency.*

### 2.5. Air Pollution Early Warning and Forecast

The most important function of air pollution early warning systems is to report the air quality to relevant departments when the air quality reaches the early warning standard. A complete pollution warning system includes the pollutant, resource, and scope of influence [44].

Air quality forecasting is an effective way of protecting public health by providing an early warning against harmful air pollutants [9]. Urban air pollution events can be forecasted by meteorological elements to provide an early warning. Therefore, in the face of more and more urban air pollution incidents, in addition to risk prevention management and emergency measures, air pollution forecasts should also include the emergency warnings as an important part of the whole emergency system.

The early warning system for air pollution is triggered before the heavy pollution of urban air, according to the forecast of meteorological elements. Corresponding emergency measures are initiated as early as possible to reduce the discharge of pollutants and mitigate the consequences. Many countries have early warning systems for pollution. For example, the Air Quality Index (AQI) value is an index for the classification of the early warning level in China, and the early warning level is determined according to the upper limit of the pollution forecast. Therefore, the forecasting of air pollution as the basis for pollution warning systems and pollution control should be highly valued by all countries.

In China, Tang believed that air pollution is different from water pollution, because serious pollution incidents may occur in a short time, if the weather situation is not conducive to the spread of pollutants [45]. However, Hong et al. showed that the potential forecast only forecasts the weather conditions of air pollution, and this method failed to give exact quantitative results for air pollution. Therefore, they developed a numerical forecasting method [46]. 

Among these various methods, there is a classic forecasting method used to forecast air pollution quantitatively, namely the AI method. Grivas et al. developed an Artificial Neural Network (ANN) combined with meteorological and time-scale input variables [22]. The input variables were selected by using a genetic algorithm optimization procedure. 

In addition, the hybrid model also developed and performed well, and widely used in various fields [47,48,49]. Güler Dincer et al. established a new Fuzzy Time Series model based on the Fuzzy K-Medoid clustering algorithm to forecast the concentration of SO_2_ in Turkey [50]. Wang et al. proposed a novel hybrid model, called Complementary Ensemble Empirical Mode Decomposition, Biogeography-Based Optimization based on Differential Evolution, and Linear Least Squares Support Vector Machine (CEEMD-BBODE-LSSVM), for air pollution point and interval forecasting [51]. Xu et al. proposed a robust early warning system that includes an evaluation module, forecasting module, and characteristics estimation module. This system defines the air quality levels and is also used to determine the main pollutants [52].

**Figure 3:**
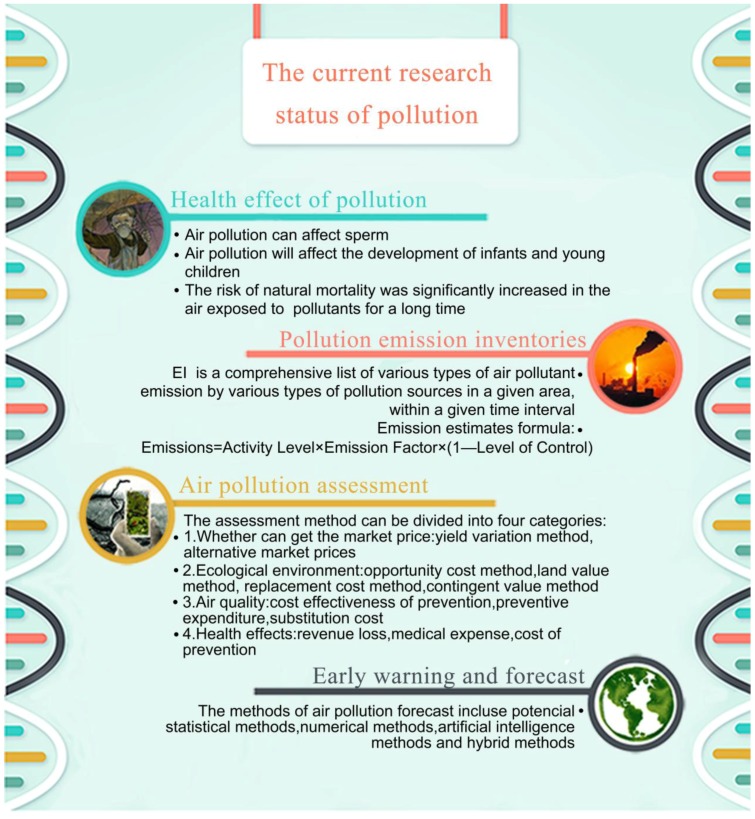
The current status of air pollution research.

In the following sections, a thorough analysis and summary of the forecasting of air pollution will be provided. The structure of the second section is clearly summarized in Figure 3.

## 3. Abbreviation Explanation and Error Assessment Index

There are many methods were descripting in our paper. And it is necessary to assessment the results of forecasting, different articles use different assessment indexes. For the convenience of reading, we give the abbreviations and used assessment indexes of various methods in the form of a list for easy reference.

### 3.1. Abbreviations

Abbreviations of methods are listed in Table 2.

### 3.2. Error Assessment Method and Index

Many performance indexes have been proposed in the field of error assessment. The definitions and formulas of the indexes involved in this study are shown in Table 3, where *F_i_* represents the forecasting value, and *A_i_* represents the actual value. 

## 4. Potential Forecasting Methods

Potential forecasting was widely used in the 1960s. It is based on combination of statistical and numerical prediction, according to certain conditions of the pollution source, the weather situation, and the meteorological index to construct the pollution potential index (PPI), and the qualitative or semi quantitative forecast of the atmospheric environment quality in the future is conducted. When the weather conditions are expected to be in line with the standards of possible serious pollution, a warning is issued [19,45].

Scott et al. applied an air pollution potential forecast model based on a synoptic climatological approach to forecast the concentration of SO_2_ in a heavily industrialized area in Durban (South Africa). Their proposed model identified periods of elevated SO_2_ successfully [53]. However, potential prediction without considering the location of pollution sources and emissions of pollutants and the accuracy of prediction is low. Therefore, more statistical models, artificial intelligence, and hybrid models are used currently.

## 5. Statistical Forecast Methods

Statistical forecast methods analyze the events without knowing the mechanism of the change; therefore, this method is not dependent on physical, chemical, or biological processes. Statistical forecasts include parametric and non-parametric statistical methods [54]. Parametric models are traditional statistical models such as linear regression and principal component analysis; nonparametric models have no defined function form. Generally, nonparametric regression includes neural network models, Gaussian process regression etc., a detailed review of the application of statistical prediction models was published in [55].

### 5.1. Regression Methods

Regression analysis is a statistical tool that investigates relationships between variables. Usually, the researchers seek to ascertain the causal effect of independent variables *Y* upon dependent variables *x_i_* [56]. When we use the model to forecast y for a particular set of values of *x_i_*, we want to measure how large the error of the forecast might be. All these elements, including dependent and independent variables and error, are part of a regression analysis, and the resulting forecast equation is often called a regression model [57]. Regression analysis is a basic technique in air pollution forecasting.

Linear regression plays a strictly utilitarian role in the field of statistical methods. Its expression is as follows:

(2) $$Y=b_{0}+b_{1}x+e$$

A multiple-linear regression (MLR) model is given as:

(3) $$Y=b_{0}+b_{1}x_{1}+b_{2}x_{2}+\cdots +b_{i}x_{i}+e$$

or:

(4) $$Y=b_{0}+\overset{n}{\underset{i=1}{\sum }}b_{i}x_{i}+e_{i}$$

where *Y* is the dependent variable, *x* and *x_i_* are the independent variables, *b* and *b_i_* are the regression coefficients, and *e* is the error. It has a normal distribution with a mean of 0.

For air pollution forecasting, *Y* represents the pollutant concentration forecast at time *t* + 1, *x_i_* represents the pollutant concentrations and meteorological variables at time *t*, *b_i_* are the regression coefficients, and *e* is an estimated error term obtained from independent random sampling. The values of *b_i_* can be obtained by using a least squares error technique [58].

Nonlinear regression analysis is an extension of the linear regression analysis, as well as the structural model of a traditional econometric analysis. In the social reality of economic life, many relationships between the analysis and forecast are generally used in nonlinear regression methods instead of a linear relationship.

In the classical regression analysis, solving the nonlinear regression problem requires the conversion of variables to a linear relationship and the use of linear regression theory to determine the regression coefficients [59]. This method has been widely used for many years in practice.

General nonlinear regression models can be written in the following form [59]:

(5) $$Y=\varphi \left(x_{1},x_{2},\ldots ,x_{m},\beta_{1},\beta_{2},\ldots ,\beta_{r}\right)+\varepsilon$$

For some special nonlinear relationships, variable transformations can be used to convert the nonlinear relationship into a linear one. The nonlinear equation can be transformed into a linear equation using the categories shown in Table 4.

Cortina-Januchs et al. used the cluster algorithm to find relationships between PM_10_ and meteorological variables and then used multilayer regression to forecast the concentration of PM_10_. The results show that meteorological variables are important in air pollution forecasting [60].

**Remark** **2.** *It should be noticed that there are many hypotheses for different regressions; and if any hypothesis is violated, the resulting estimate is biased. Therefore, the availability of regression methods should be taken into full consideration in solving exact problems. Moreover, in order to improve the prediction accuracy of regression equations, researchers often increase the variables in the regression equation. However, the increase of independent variables will increase the calculations. The regression process becomes longer, and the prediction problems and control problems become complicated. Therefore, the main problem of the regression model is to choose the variables for the regression equation. This requires significant experimental investigation* *.*

### 5.2. ARIMA Methods

The autoregressive integrated moving average (ARIMA) model is a linear model that can show steady state in both stationary and non-stationary time series. When constructing the ARIMA model, there are three main steps (Rahman et al. [21]):

***Step 1*.** Tentative identification

***Step 2*.** Parameter estimation

***Step 3.*** Diagnostic checking

ARIMA with a seasonal difference is called SARIMA. SARIMA processes the data with a seasonal period length *S*; and if *d* and *D* are non-negative integers, the difference series, *W_t_* = (1 − *B*)d(1 − *B*^5^)*^D^x_i_*, is a stationary autoregressive moving average process [61]. The SARIMA model can be written as:

(6) $$\phi_{p}\left(B\right)\phi_{p}\left(B^{S}\right)W_{t}=\theta_{q}\left(B\right)\Theta_{Q}\left(B^{S}\right)\varepsilon_{t}\;\;\;\;\;\;\;\;\;\;\;t=1,2,\;\ldots ,\;N$$

where *N* is the number of observations up to time *t*; *B* is the backshift operator defined by *B^α^W_t_* = *W_t−__α_*; *ϕ_p_*(B) = 1 − *ϕ*_1_*B − … −* *ϕ_p_B_p_* is called a regular (non-seasonal) autoregressive operator of order *p*; *ϕ_p_*(B*^s^*) = 1 − *ϕ*_1_*B^s^* − *…* − *ϕ_p_B^ps^* is a seasonal autoregressive operator of order *p*; *θ_q_*(B) = 1 − *θ*_1_*B* − *…* − *θ_q_B^q^* is a regular moving average operator of order *q*; Θ*_Q_*(B*^S^*) = 1 − Θ_1_*B^S^* − *…* − Θ*_Q_B^QS^* is a seasonal moving average operator of order *Q*; *ε_t_* is identically and independently distributed as normal random variables with mean zero, variance *α*^2^ and cov(*ε_t_*, *ε_t_*_−*k*_) = 0, ∀k ≠ 0 [61].

In the definition above, *p* represents the autoregressive term; *q* is moving average order; *P* represents the seasonal period length of the model, *S*, of the autoregressive term; *Q* represents the seasonal period length of the model, *S*, of moving average order; *D* represents the order of seasonal differencing; and *d* represents the order of ordinary differencing [61].

When fitting a SARIMA model to data, the estimation of the values of *d* and *D* is primary, with the orders of differencing needed to make the series stationary and to remove most of the seasonality. The values of *p*, *q* and *Q* need to be estimated by the autocorrelation function (ACF) and partial autocorrelation function (PACF) of the differenced series and other parameters can be estimated by suitable iterative procedures [61].

Rahman et al. (2015) forecasted the API from three different stations [21]. The forecasting accuracy of the possible SARIMA model is shown in Table 5.

In this study, the authors contrasted the result of SARIMA and a fuzzy time series (FTS) model. According to the result, the conventional ARIMA model outperformed the FTS model in two urban areas and the FTS only perform better in a sub-urban area.

**Remark** **3.** *The ARIMA model requires time series data to be stable or stable after differentiation. Moreover, the ARIMA model can only describe the linear relationship between variables to model and predict and cannot describe the nonlinear relationship between variables. However, pollution data are complex and combine geography, weather, and other factors to make data unstable and nonlinear, so the data should be processed into a stable and linear format before forecasting by ARIMA. If the data cannot be processed into stable and linear, other forecasting models should be chosen.*

### 5.3. Projection Pursuit Model (PP)

This method was developed in the 1970s. The main idea of air pollution forecasting statistical methods is to be a “supposition-simulation-forecast”, so those methods are not suitable for analyzing the data of nonlinear relationships or non-normal distributions. In contrast, the projection pursuit (PP) technique presents a new method of exploratory data analysis of “review of data-simulation-forecast”, which can be used to a certain extent in some nonlinear problems [62]. The main idea of projection pursuit is to machine-pick low dimensional projections of high dimensional point cloud by numerically maximizing a certain objective function or projection index [63].

The general form of an order *K* PP autoregression model is as follows:

(7) $$x_{i}=\overset{M}{\underset{m=1}{\sum }}\beta_{m}G_{m}\left(Z_{m}\right)$$

(8) $$Z_{m}=a_{m_{1}}x_{t-1}+a_{m_{2}}x_{t-2}+\cdots +a_{m_{k}}x_{t-k}$$

where Z*_m_* is the estimated value of time series {*x*} at *t* time; *x_i_* represents *K* time series forecast factors, its selection is ultimately determined by the data structure; *a_m_* represents the projection direction for the *m*th content, it satisfies $\|a_{m}\|=1$; *G_m_* is the optimal piecewise linear function of Z*_m_*, called ridge function. It is a numerical function; *β_m_* is the weight coefficients of the contribution of the *m*th ridge function to *X_t_*.

The optimization process of the final model can be divided into two steps [6]:

***Step 1***. Local optimization process

The highest linear combination of *M* and the optimal parameters *α_m_* and *β_m_*, and the ridge functions *G_m_* are determined by the stepwise alternating optimization method.

***Step 2***. Global optimization process

In order to find a better model, the linear combination of *M* and the number of parameters were optimized further, eliminating the unimportant items in the model one by one. The model number decreased to *M_u_*,*M_u−_*_1_,L,1, determined the number for *M*, and found the best solution of the minimum *M*.

Deng et al. (1997) used PP regression to forecast SO_2_ concentration based on historic data [62]. 

At first, standardizing SO_2_ concentration data according to Equation (9):

(9) $$C_{i}^{1}=\frac{C_{i}-C_{min}}{C_{max}-C_{min}}$$

The range of $C_{i}^{1}$ values is listed in Table 6:

Sample test results are shown in Table 7.

The authors defined that when the absolute relative errors were less than 20%, the result was qualified; therefore, in their study, the forecast accuracy of the sample was 75%.

**Remark** **4.** *The PP method overcomes the difficulties of the “dimensionless curse” caused by high dimensional distribution and has the advantages of assumption, objective, robustness, anti-interference, accuracy, wide applicability, and rapid modeling. It can adapt to the form of flexible development requirements. For different research objects, it can use various forms of the model based on this method. Therefore, a series of methods, such as the PP regression and PP clustering methods, have been derived. However, this method also has many disadvantages, including complex computation, difficulties in finding the optimal projection direction, falling into local optima easily, and difficulties in solving highly nonlinear problems.*

### 5.4. Principal Component Analysis Model

A principal component analysis (PCA) is a multivariate statistical analysis technique based on data compression and feature extraction. PCA is able to extract the dominant patterns in the matrix in terms of a complementary set of score and loading plots. And those extracted patterns contain majority information of the original data [64].

A PCA reduces the number of predictor variables by transforming them into new variables; those new variables are called principal components (PCs). These PCs retain the maximum possible variance of the same data. The correlation matrix of the normalized input data can provide the PCs, and the eigenvalues of the correlation matrix “*C*” are obtained from its characteristic equation as given in Equation (10) [25]:

(10) |c−λI|=0

where *λ* is the eigenvalue, and *I* is the identity matrix. For every eigenvalue, there is a non-zero eigenvector, which can be defined as:

(11) $$c_{e}=\lambda e$$

The *i*th variance of the *i*th PC is given as:

(12) $$Variance=\frac{\lambda_{i}}{\sum_{n}\lambda_{n}}$$

After obtaining all of the PCs, the initial data set is transformed into the orthogonal set by multiplying the eigenvectors [58,65].

Kumar et al. (2011) proposed a PCR model to forecast AQI in Delhi. The so-called PCR model transformed the data set into a multiple linear regression equation [58].

**Remark** **5.** *The PCA algorithm reduces the dimensions of a series. It converts a number of related variables into a small number of unrelated variables that contain large amounts of original information. In the application of PCA, we choose the index to be representative, objective, independent, and comprehensive. At the same time, if the data set contains extreme values and nonlinear variables, the analysis effect will be greatly discounted. Therefore, the nonlinear PCA and independent component analysis methods are proposed, and these two methods are widely used in the forecasting field, but they are rarely used in pollution forecasting and need to be further explored.*

### 5.5. Support Vector Regression

Support vector regression (SVR) is the application of support vectors in a regression function. There are two main types used for the regression analysis in SVR: *ε*-*SVR* and *ν*-*SVR*. SVR have advantages in high dimensionality space because SVR optimization does not depend on the dimensionality of the input space [66].

In the highly dimensional feature space, there is a linear function, which maps the input data into higher dimensional space through nonlinear mapping. Such a linear function is known as the SVR equation [24]:

(13) f(x)=(w×φ(x))+b

where *f*(*x*) indicates forecast value; *w* is N-dimensional weight vector; the dimension of *w* is the dimension of feature space; *b* is the threshold. The specific calculation method of (*w*,*b*) is given in [26].

Chen et al. used SVR to forecast the concentration of SO_2_. First, they analyzed and forecasted the influencing factors. Next, as a key step, they preprocessed the daily average concentration of SO_2_, covering the period during 2001–2002 in Xi’an by using PCA to reduce the dimensionality of the input factors. Finally, the support vector regression model based on the radial basis function (RBF) kernel was established [67].

**Remark** **6.** *Statistics are widely used in the forecasting field, and many existing models are based on it, such as the support vector machine (SVM). However, there are some problems in the application of classical statistical forecasting. For example, forecasting results from a single model are worse and have a low degree of integration with other methods. Therefore, researchers improve the statistical forecasting methods through various channels, such as proposing new hybrid models, changing the form of input variables, and studying new criteria for error evaluation. These measures have improved the prediction accuracy to varying degrees.*

### 5.6. Artificial Neural Network 

An ANN is a NN that mimics animal behavior characteristics. It is a mathematical model of distributed parallel information processing. ANN relies on the complexity of the system, through adjustment of the internal connection between a large numbers of nodes, to achieve the purpose of processing information. The NN has the capabilities of self-learning and self-adaptation.

A common feed forward Network Multilayer consists of three parts: the input layer, hidden layer, and output layer, and each of the layers contains several processing units connected by acyclic links. Those link points are named neurons.

From the viewpoint of mathematics, the hidden neuron *h_j_* can be described by the Equation (14) [68]:

(14) $$h_{j}=\varphi \left(z_{j}\right)$$

where *ϕ*(*z_j_*) is an activation function, usually expressed as $z_{j}=\sum_{i=l}^{l}w_{ij}x_{i}+b_{j}$; $\varphi \left(x\right)=\frac{1}{1+e^{-x}}$; *w_ij_* is the weight of input *x_i_* at neuron *j*; *b_j_* represent bias of neuron *j*.

The relationship between the output *f*(*x*) and the inputs has the following representation:

(15) $$f\left(x\right)=w_{0}+\overset{q}{\underset{j=1}{\sum }}w_{j}h_{j}$$

where *w_j_* is a model parameter, often called connection weights; *q* is the number of hidden nodes.

An ANN is representative of AI methods for forecasting air pollution. Wang et al. (2015) used an ANN model to forecast the concentrations of SO_2_ and PM_10_ in four stations in Taiyuan to compare with a hybrid model. The ANN forecast accuracy is shown in Table 8 [68].

In Rahman’s study, they contrasted the results of SARIMA, ANN, and a fuzzy time series (FTS), and the results are shown in Table 9. The study results indicated that the ANN model was capable of modeling and forecasting index values of API [21].

Elangasinghe et al. built an ANN air pollution forecast model based on meteorological parameters and the emission patterns of the sources. First, they identified the various data sets, and after cleaning, normalizing, and randomizing the data, they built an ANN model. Then, they applied forward selection, backward elimination, and genetic algorithms with sensitivity analysis techniques as the selection tool to eliminate the irrelevant inputs from the network [23].

**Remark** **7.** *The ANN, as the simplest NN, has been applied to predict air pollution. It has good nonlinear fitting ability and improves the prediction accuracy. However, there are many factors affecting pollution and the relationship is complicated for clarifying the relationship between these factors and improving the prediction accuracy of the ANN.*

### 5.7. Back Propagation Neural Network

Back propagation (BP), meaning “error backward propagation”, is one of the most widely used NN models, which is trained by the error back propagation algorithm. It consists of two processes: the forward propagation of information and the back propagation of error. When the actual output is not in conformity with the expected output, the reverse propagation phase of the error is entered. The error is corrected by the output layer, and the weight of each layer is updated by the error gradient descent method. The cycle of information forward propagation and error back propagation processes and the constant adjustment of the weights of each layer are the learning and training processes of the NN, and those two processes are executed until the network output error is reduced to an acceptable level or pre-set learning times are reached.

When modeling a BPNN, the number of hidden nodes is the primary variable to be determined. Recently, the trial and error method and an empirical formula (Equation (16)) have been applied to solve this issue [24]:

(16) $$hidden\;nodes=\sqrt{m+p}+a,a\in \left[0,10\right]$$

Bai et al. improved the BPNN model based on wavelet decomposition to improve the feature representations in multi-scales and weaken the randomness. The operations of the model are as follow [24]:

***Step 1:*** Collect the modeling data that contain historical air pollutants concentrations C and meteorological data M.

***Step 2:*** Perform the stationary wavelet transform (SWT) to decompose the time series of C.

***Step 3:*** Normalize the meteorological parameters and one level of wavelet coefficients into [0, 1] according to Equation (17):

(17) $$Normalization=\frac{data-data_{\min}}{data_{\max}-data_{\min}}$$

***Step 4:*** Calculate the *t*th wavelet coefficients of the *z*th scale using *BPNN_z_*, *z =* 1, 2, …, *l*, *l* + 1 with the *t*th meteorological data and (*t* − 1)th wavelet coefficients:

(18) $$\{\begin{array}{c} D_{n}\left(t\right)=BPNN_{n}\left(M\left(t\right),\;D_{n}\left(t-1\right)\right)n\in \left[1,l\right]\\ A_{l}\left(t\right)=BPNN_{l+1}\left(M\left(t\right),\;A_{L}\left(t-1\right)\right) \end{array}$$

***Step 5:*** Perform the inverse SWT to generate the estimated daily pollutants concentrations.

(19) $$c\left(t\right)=ISWT\left(D_{1}\left(t\right),D_{2}\left(t\right),\ldots ,D_{l}\left(t\right),A_{l}\left(t\right)\right)$$

***Step 6:*** Output the forecasting result.

The comparison between the results of W-BPNN and BPNN are shown in Table 10. From the table, we find that the values of the mean absolute percent error (MAPE) and root mean square error (RMSE) for W-BPNN are lower than the values for BPNN, which indicates that W-BPNN has the best forecasting performance.

Wang et al. improved the BPNN from other side. They added SSA algorithm to reduce the effect of chaotic nature on pollution sequences and improve BPNN forecasting performance [69].

**Remark** **8.** *The convergence speed of the BPNN is slow, and it cannot guarantee the convergence to the global optimum. At the same time, the selection of the operational parameters of the BPNN is generally based on experience and lacks theoretical guidance. Therefore, when using BP, it should be combined with other optimization algorithms to improve its prediction accuracy.*

### 5.8. Wavelet Neural Network

Another commonly used NN is the wavelet NN. As the name suggests, the term wavelet means a small waveform, and “small” means that it has decay, and ”wave” refers to its volatility. Wavelet analysis is used to gradually refine the signal (function) through expansion and translation operations. Finally, the high frequency is subdivided by time, and the low frequency is subdivided by frequency. A wavelet analysis can automatically adapt to the requirements of a time-frequency signal analysis, so it can focus on any details of the signal.

The wavelet function *ψ*(*t*) refers to a shock characteristic that can quickly decay to zero for a class of functions, defined in Chen [70] as:

(20) $$\int_{-\infty }^{+\infty }\psi \left(t\right)dt=0$$

If *ψ*(*t*) satisfies the following admissibility condition (Equation (21)), we term *ψ*(*t*) as a basic wavelet or wavelet:

(21) $$c_{\psi }=\int_{-\infty }^{+\infty }\frac{{\left|\psi \left(t\right)\right|}^{2}}{\omega }<\infty$$

After dilation and translation of function *ψ*(*t*), we obtain Equation (22):

(22) $$W_{f}\left(a,b\right)={\left|a\right|}^{-\frac{1}{2}}\psi (\frac{t-b}{a})$$

This is called a wavelet sequence, where *a* is the expansion factor, and *b* is the translation factor.

The wavelet transform of the function *f*(*t*) is as follows:

(23) $$W_{f}\left(a,b\right)={\left|a\right|}^{-\frac{1}{2}}\int_{-\infty }^{+\infty }f\left(t\right)\psi (\frac{t-b}{a})\mathrm{dt}$$

where *w_f_*(*a,b*) are the wavelet coefficients, which can reflect the characteristics of the frequency domain parameter a and the time domain parameter *b*. When parameter *a* is smaller, the resolution of the frequency domain is lower, but the resolution is higher in the time domain. In contrast, when *a* is larger, the resolution of the frequency domain is higher, and the resolution is lower in the time domain. Therefore, the wavelet transform can realize the time frequency localization of the fixed size and variable shape of the window.

Chen applied the method of wavelet analysis and neural networks to forecast the concentration of atmospheric pollutants. The steps in the study were [70]:

***Step 1:*** The low frequency coefficients of the highest layer are reconstructed after wavelet decomposition, clearly determining the annual change of atmospheric pollutant concentration. By using wavelet decomposition, the lowest two layers with high frequency signals are reconstructed, so abrupt change points of the time series of atmospheric pollutant concentration can be clearly judged.

***Step 2:*** The time series of atmospheric pollutant concentration are decomposed into different frequency channels by wavelet decomposition, and then the corresponding time series model is considered. Finally, the predicted values of different frequency channels are combined to obtain the predictive value of the original time series.

***Step 3:*** The input samples of the NN prediction model are studied, and the input variables of the NN prediction model are analyzed by using the principle of atmospheric pollution meteorology. Then, the PCA is used to reduce the dimension of the input variables.

***Step 4:*** The annual variation trend of atmospheric pollutant concentration time series are segmented by wavelet decomposition and reconstruction. On this basis, the NN prediction model is designed for each segment.

***Step 5:*** The decomposed wavelet coefficients are reconstructed to the original scale, and the NN that contains the meteorological elements is applied to analyze the wavelet coefficients of low and medium frequency. For the high frequency wavelet coefficients, the wavelet coefficients of the first few days are used as the input values of the NN model. Finally, the forecast of each wavelet coefficient sequence value is synthetized, and the forecasted value of the original sequence is obtained.

A summary of commonly used wavelet is shown in Table 11.

**Remark** **9.** *Wavelet analysis is proposed to overcome the shortcomings of the Fourier transform in analyzing non-stationary signals, and it can effectively extract the local information of signals and has good analysis ability for the localization time-frequency. However, the selection of the wavelet basis is difficult.*

ANNs have the advantages of self-organized learning and adaptive and good fault tolerance; however, the traditional NNs also have some shortcomings, such as slow convergence, and they easily fall into local minima. Therefore, Zhang et al. proposed the concept and algorithm for a wavelet NN in 1992. Wavelet NNs inherit the merits of a wavelet analysis and NN and function well for of approximations and for their pattern classification ability; therefore, they are widely used in prediction [71].

### 5.9. Support Vector Machine (SVM)

Support vector machine (SVM) is a new generation of machine learning technology based on statistical learning theory developed by Vapnik, and practical problems, such as small samples, nonlinearity, high dimensions, and local minimum points, can be solved effectively. SVM is successfully used in classification, regression and time series forecasts, and other fields. Originally, SVMs were used for classification purposes, but their principles can be extended easily to the task of regression and time series forecasting [61].

Forecast models of atmospheric pollutant concentration change based on SVM. The key issues are the determination of the input mode, the selection of training samples, and the selection of model structure parameters [72]. The steps to build an atmospheric pollutant concentration forecast model are as follows:

1. Build an effective forecast factor.
2. Select kernel function and parameter values.
3. Train the sample to provide the SVM forecast model with optimized parameters, get the support vector, and then determine the structure of the SVM.
4. Train the support vector predictor to forecast the test samples.

Chen used SO_2_ as an example and established a forecast model for atmospheric pollutant concentration. The author chose different kernel functions to analyze and compare each function’s mean relative error (MRE) and RMSE. Ultimately, studies showed that different kernel functions have different prediction results. They established the model that combined wavelet decomposition with SVM to forecast urban atmospheric pollutant concentration [67]. Wang et al. improved the forecast accuracy of SVM by using the Taylor expansion forecasting model to revise the residual series [68]. The forecast accuracies are shown in Table 12.

**Remark** **10.** *SVMs were initially used as a pattern recognition method based on statistical learning theory and has better predictive processing ability for small samples and nonlinear data. However, the SVM algorithm is difficult to be implemented in large-scale training samples. When the number of training samples is large, the storage and computation of the data matrix will consume a great deal of machine memory and computation time. At the same time, SVMs are sensitive to missing data. If there are more missing values in the data sequence, the accuracy of the forecasting results will be affected. SVMs have no general solutions to nonlinear problems. Since the choice of kernel function is the key to solving problems, the kernel function should be carefully chosen. In addition, the SVM algorithm only gives a two-class algorithm; there are limitations on the solution of multi-classification problems.*

### 5.10. Fuzzy Time Series (FTS) Analysis 

In 1993, Chissom and Song proposed the definition of fuzzy time series (FTS) based on fuzzy set [73]. At present, FTS has been used in the field of air pollution forecasting. The method for forecasting the API using the FTS simply can be presented as follows [21,74]:

***Step 1:*** Define and partition the universe of discourse U = (D_min_ − D_1_, D_max_ + D_2_) into several equal intervals denoted as u_1_,u_2_,L,u_m_.

***Step 2:*** Based on the SARIMA model, determine the FLRs. 

***Step 3:*** In order to select the best input for FLR, different combination inputs are attempted from single input to two inputs, three inputs and four inputs.

***Step 4:*** The optimum length of intervals was calculated by following the average-based length.

***Step 5:*** The forecasted outputs are calculated.

Rahman et al. forecasted the API for three different stations in Malaysia [21]. The forecasting accuracy in the testing period of the FTS model is shown in Table 13. 

### 5.11. Fuzzy Recognition 

**Figure 4:**
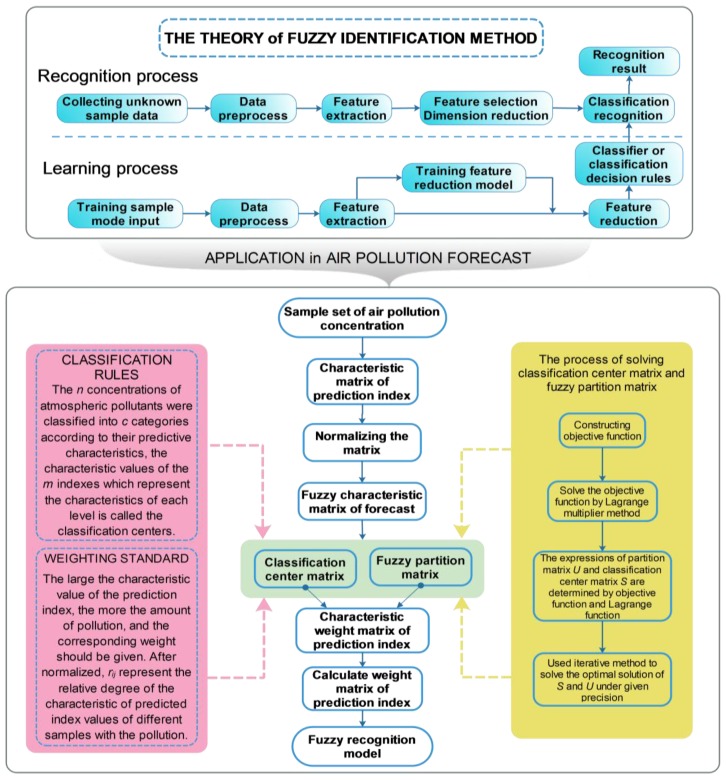
The flaw chart of fuzzy identification.

Fuzzy pattern recognition recognizes a given object, and there are usually two processes in fuzzy identification: the recognition process and the learning process. Figure 4 shows the steps for the two processes.

Xiong et al. proposed the fuzzy recognition theory and model for air pollution concentration forecasting and made an empirical study based on the measured concentration data of SO_2_. Fuzzy recognition can be used to forecast the information [75]. The forecast model contains the index weight matrix, which provides a new way of improving the forecast accuracy.

**Remark** **11.** *Theoretically, fuzzy methods have a high fault tolerance and do not require accurate mathematical models with each man-machine interaction; however, those methods have a relatively low accuracy and lack systematization. The computation of fuzzy identification is complex, and the performance of the fuzzy time series forecasting method is affected when outliers exist. The fuzzy method should be further optimized during its application, for example, combining subtractive clustering, optimizing the parameters of membership functions, and combining a BPNN to optimize fuzzy rules. The quantization factor and proportionality factor are optimized based on genetic algorithms.*

### 5.12. Adaptive Neural Network Fuzzy Inference System

ANFIS is a fuzzy inference system based on adaptive networks structure, it uses neural network algorithms to obtain fuzzy rules and membership functions from data, and uses neural networks to implement fuzzy inference processes. The general principles and methods of ANFIS have been systematically studied and summarized in the Ref [76]. ANFIS is composed of two parts: primary and inference. These two parts are connected by a network with fuzzy rules [77]. 

The most commonly used ANFIS structure is the Sugeno fuzzy model. The basic structure can be expressed as a feedforward NN with five layers [78]:

***Layer 1*:** In this layer, every node *i* is an adaptive node and the node function is the membership function to determine the degree of satisfaction. All the parameters in this layer are called antecedent parameters.

(24) $$o_{i}^{1}=\mu_{A_{i}}\left(x\right)$$

where *x* is the input to node *i*, *A_i_* is a linguistic label to node *i*, and $o_{i}^{1}$ is the membership grade of *A_i_*.

***Layer 2*:** Every node in this layer is a circle node labeled $o_{i}^{2}$ and the output is the multiplies of all incoming signals [79]:

(25) $$o_{i}^{2}=\omega_{i}=\mu_{A_{i}}\left(x\right)\times \mu_{B}\left(y\right)$$

***Layer 3*:** The output of every node *i* is called normalized firing strength. Each node calculates the rate of the *i*th rule’s firing strength to the sum of all the rules’ firing strengths and normalization [78]:

(26) $$o_{i}^{3}={\bar{\omega }}_{i}=\frac{\omega_{i}}{\omega_{1}+\omega_{2}}$$

***Layer 4*:** This layer is the conclusion layer, every node *i* is a square node or adaptive node with a node function. And parameters in this layer will be referred to as consequent parameters [79].

(27) $$o_{i}^{4}={\bar{\omega }}_{i}f_{i}={\bar{\omega }}_{i}(p_{i}x+q_{i}y+r_{i})$$

where ${\bar{\omega }}_{i}$ is the output of Layer 3 and (*p_i_,q_i_,r_i_*) is the parameter set of this node. 

***Layer 5*:** In this layer, the single node is a fixed node that computes the summation of all incoming signals [77].

(28) $$o_{i}^{5}=\underset{i}{\sum }{\bar{\omega }}_{i}f_{i}=\frac{\sum_{i}\omega_{i}f_{i}}{\sum_{i}\omega_{i}}$$

**Remark** **12.** *It is being proven that the accuracy of AI forecasts is higher than traditional statistical forecasts. More recently, researchers select meteorological or geographic factors as input variables, and those adjusted models are shown to improve the accuracy of forecasting. From the results of a comparison by Rahman (2015), ANN can be used to predict the fluctuation series, which contain certain trends and seasonality, such as those in air quality data. However, ANN and SVM have limitations inherent to their input variables; their main defect is a failure to obtain complete information about research questions related to learning goals. Therefore, the shortcomings of ANNs facilitate the development of hybrid models.*

## 6. Three Dimensional Models

Over the past few years, studies of air pollutants concentration forecast have been expanded from two-dimensional space to three-dimensional space. Compared to two dimensional models, three-dimensional models are more accurate when addressing complicated terrain, boundaries, etc. Three dimensional models take the movement of pollutants in the horizontal and vertical directions into account, and are more consistent with actual emission conditions, so that the distribution of pollutants concentration is more realistic. A detailed review of three dimensional model can be found in Ref [80]. Here we mainly introduce some representative models.

### 6.1. Emissions Methods

The atmospheric emissions model is also known as the air quality model. It has undergone three generations of development and improvement in the past 50 years. 

The first generation of air quality model mainly includes the box model based on the mass conservation law, the Gaussian model based on the turbulence diffusion statistical theory, and the Lagrange trajectory model [81]. The first generation models had no or only simple chemical reaction modules, which limits their application in practice. However, these models are still widely used in the simulation of conventional pollutants due to their simple structure, fast calculation speed, and high accuracy of long-term concentration simulation.

From the early 1980s to the early 1990s, advanced in the study of physics and atmospheric chemistry mechanisms of clouds and precipitation, the air quality model has been correspondingly developed, in which more complex meteorological models, meteorological parameters, and detailed nonlinear chemical reaction mechanisms are added. Gradually, the second-generation air quality model based on the Euler grid model was formed.

Since the 1990s, in order to consider all of the atmospheric problems, the third generation air quality model based on the so-called “one atmosphere” was concept. The “One atmosphere” use the entire atmosphere as a research object, simulating all atmospheric physics and chemical processes at various spatial scales. The current mainstream models include CMAQ, Comprehensive Air Quality Model with Extensions (CAMx), and Weather Research and Forecasting Model coupled with Chemistry (WRF-Chem) etc.

At present, there are two main calculation methods of emission model:

(1) Air quality forecasting based on statistical methods. They use statistical methods to analyze existing data, explore changes in the atmospheric environment, and predict concentrations of air pollutants by establishing statistical forecast models between air pollution concentrations and meteorological parameters.
(2) Numerical forecasting based on atmospheric dynamics theory. These methods are based on the understanding of the physical and chemical processes of the atmosphere and use computers to forecast the dynamic distribution of air pollutants concentrations by establishing a numerical model for the transport and diffusion.

#### 6.1.1. The Atmospheric Dispersion Modelling System 

The Atmospheric Dispersion Modelling System (ADMS) is developed by the Cambridge Environment Research Corporation (CERC) and is currently one of the mainstream models of international atmospheric diffusion. The ADMS model uses a three-dimensional Gaussian model to calculate the concentration of pollutants with a Gaussian distribution, and to consider the distribution of pollutants under neutral and unstable conditions. 

ADMS needs to input the pollution source intensity data, meteorological condition data and topographic data of calculation point. The output includes the average concentration of contaminants at a certain point or area, and the average time ranges from 10 min to the annual average. The output also includes dry and wet deposition, and radioactivity effects. In addition, according to the air quality standard, the number of violations can also be calculated. At the same time, the meteorological preprocessing module will also generate an output file of meteorological data, the data is different from conventional meteorological data.

Riddle et al. compared atmospheric diffusion modeling capabilities between ADMS and another model which was based on computational fluid dynamics. The results showed that ADMS performed better under neutral stability, due to its fast calculation speed and simplicity in model setting up [82].

Manar et al. coupled the regional mesoscale model WRF and local scale ADMS to structure a one-way coupled modeling system WRF-ADMS. The main function of ADMS was to model fast atmospheric stability resolving and turbulence with Gaussian dispersion model. And in their study ADMS was driven by WRF in an offline mode [83]. In this system, the authors input large scale weather data (resolution initial and boundary conditions), and static data (resolution topography, resolution land use and soil data) to WRF framework, and then output three dimensional weather forecast predictors. And the input data of ADMS was very fine grid data, consisting of detailed characteristics of the stack properties, hourly averaged meteorological data, mesoscale wind filed, sensible heat flux and boundary layer height etc. And the output from ADMS system are wind/turbulence flow filed data and pollutants concentrations/depositions etc. The experimental results indicated that ADMS model allowed for simulations with the mesoscale forecast. And WRF-ADMS dispersion modeling system could simulate the observed sptial distributions of Perfluoromethylcyclohexane plumes [83].

#### 6.1.2. The California Puff Model

The California Puff model (CALPUFF) is an unsteady three-dimensional Lagrangian puff transport and dispersion model that simulates the discrete and transform processes along the advects “puff” of matter emitted from model source. Puff mode is a relatively simple and flexible diffusion mode that can deal with severe weather conditions and pollution source parameters of variation in temporal and spatial and is more widely used than Gaussian plume mode.

Tartakovsky et al. calculated and forecasted the concentrations of particulate emissions from a quarry located in hilly terrain by CALPUFF and AERMOD (the American Meteorological Society—United States Environmental Protection Agency (US EPA) Regulatory Model). They compared the result of two models, and the result indicated that when the terrain was simple, and the data was good, the CALPUFF performed better. Moreover, the model was more sensitive to the quality of the meteorological data [84].

**Figure 5:**
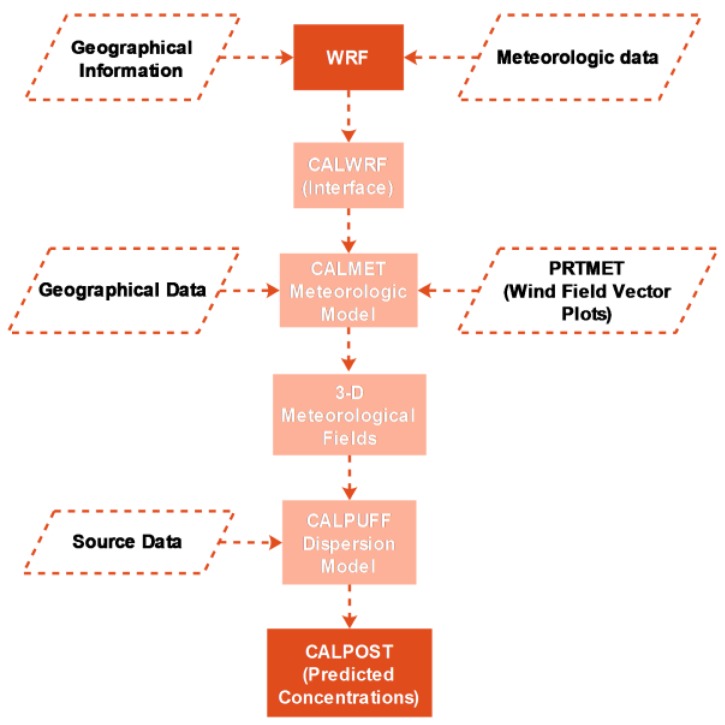
The process diagram of WRF–CALMET–CALPUFF modeling system.

Abdul-Wahab et al. coupled the CALPUFF and WRF to investigate the transport and dispersion patterns of SO_2_ from refinery in Oman, and to forecast the concentration of SO_2_. The initial phase in their study was to input meteorological data and geographical information to WRF in order to obtain meteorological fields for CALWRF (an interface program). Then, the second phase was to input meteorological fields generated from CALWRF and geophysical data to California Meteorological Model (CALMET). The final step was to extraction meteorological parameters from CALMET output file, and put those meteorological parameters into CALPUFF dispersion model to get predicted concentrations [85]. The process of WRF-CALMET-CALPUFF model is shown in Figure 5.

Falke et al. designed a system to enable the fire location, forecast smoke and calculates population statistics. They initialize a CALPUFF smoke model by using fire locations derived from satellites and surface observations and reconciled through US forecast service. Forecasting results were used to obtain population information that was expected to be affected by wildfire smoke, by combining with web service. These population information can be used to conduct preventive work [86]. 

#### 6.1.3. CMAQ Model

CMAQ was designed from the start as a community model. “Community modeling” refers to the concept that air quality model development should be a collective effort by a broad community of developers. For more details, please visit the website, https://www.cmascenter.org/cmaq/.

The CMAQ consists of several processors and chemical-transport models:

Meteorology-chemistry interface processor (MCIP)
Photolysis rate processor (JPROC)
Initial conditions processor (ICON)
Boundary conditions processor (BCON)
CMAQ chemical-transport model (CCTM)

The relationship of the modules is described as follows:

(1) The core of the CMAQ is the chemical transport module CCTM, and it can simulate the transport process, chemical process, and sedimentation process of pollutants.
(2) The initial module ICON and the boundary module BCON provide the initial field and boundary field of pollutants for CCTM.
(3) The photochemical decomposition rate module JPROC calculates the photochemical decomposition rate.
(4) The meteorological chemical interface module is the interface between the meteorological model and CCTM, and it can transform meteorological data into a CCTM identifiable data format.

The CCTM module can be extended, for example, to add a cloud process module, diffusion and transfer module, and aerosol module in this module. The operator can choose to add these modules in CMAQ in order to facilitate the simulation of the model in different regions. The meteorological field needed by the numerical calculation of CMAQ is provided by the meteorological models, such as the Mesoscale Meteorological Model 5 (MM5) and the Weather Research and Forecasting (WRF) Model. The required emission inventory is provided by an emission processing model, such as the Sparse Matrix Operator Kernel Emissions (SMOKE). But in MM5 version 3, output will be able to be processed by MCIP, and the MCIP is no longer needed with SMOKE [87]. CMAQ requires two primary types of inputs: meteorological information and emission rates from sources of emissions that affect the air quality [27].

**Figure 6:**
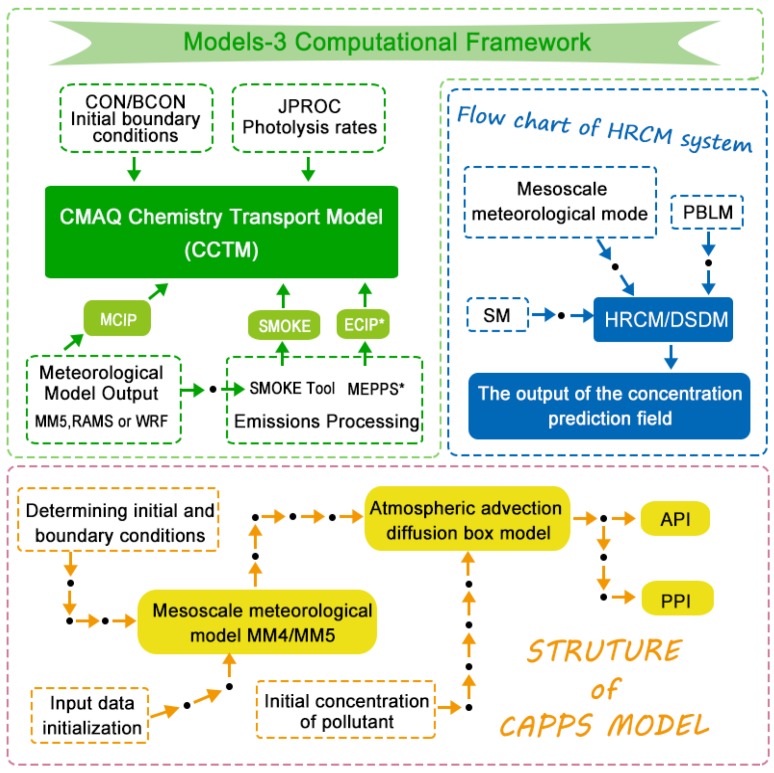
Structures of numerical forecast methods.

Model-3/CMAQ is made up of three parts: CMAQ, MM5, and SMOKE. CMAQ is the core, and MM5 and SMOKE provide the necessary data. In this model, the meteorological background field provided by MM5 is developed first, and then the Meteorology-Chemistry Interface Processor (MCIP) is used to deal with the meteorological field and to provide data to CMAQ and SMOKE. The relationship between MM5, CMAQ, and SMOKE is shown in Figure 6.

Glahn et al. put forward the model output statistics method (MOS) [88]. The CMAQ-MOS model uses early atmospheric monitoring data combined with the data of CMAQ, which can correct the deviation of the air quality forecast caused by the subjective pollutant emission source. The CMAQ-MOS model needs the atmospheric monitoring data of the meteorological elements and multiple pollutants.

The CMAQ provides the output of multiple pollutants using the CMAQ model. The mathematical expression of CMAQ-MOS is as follows [65]:

(29) $$S^{n}={\hat{G}}_{m}+{\hat{S}}_{M+N+L}\times {\hat{A}}_{m}^{M+N+L}$$

where *S^n^* is the forecast object which represents the concentration of a particular pollution on the forecasting day (the *n*th day); the (*n* − 1)th day is the initial day; ${\hat{G}}_{m}$ and ${\hat{A}}_{m}^{M+N+L}$ are coefficient matrices; m is time stage and ${\hat{S}}_{M+N+L}$ has three forecast factors represented as follows [65]:

$${\hat{S}}_{M+N+L}=\left(\begin{array}{c} X_{L}\\ Y_{M}\\ Z_{N} \end{array}\right)$$

(1) $X_{L}=\left(\begin{array}{c} x_{1}^{n-1}\\ x_{2}^{n-1}\\ \ldots \\ x_{L}^{n-1} \end{array}\right)\;$represents the monitoring values of *L* types pollutant concentrations on the initial forecasting day.
(2) $Y_{M}=\left(\begin{array}{c} y_{1}^{n-1}\\ y_{2}^{n-1}\\ \ldots \\ y_{M}^{n-1} \end{array}\right)\;$represents the monitoring values of *M* types atmospheric elements on the initial forecasting day.
(3) $Z_{N}=\left(\begin{array}{c} z_{1}^{n}\\ z_{2}^{n}\\ \ldots \\ z_{N}^{n} \end{array}\right)\;$represents the forecast values of *N* types of pollutant concentrations on the forecasting day.

The variables for CMAQ-MOS experiment include the wind field (U, V), temperature field (TS), and relative humidity (RH).

Zhao et al. used the information above to build equations for 12 observation sites to forecast the concentrations of PM_2.5_, PM_10_, SO_2_, NO_2_, and O_3_ in Tianjin [65].

$$PC=g+\;a_{1}\times {\left[PM_{10}\right]}_{1}+a_{2}\times {\left[SO_{2}\right]}_{1}+a_{3}\times {\left[NO_{2}\right]}_{1}+a_{4}\times {\left[PM_{10}\right]}_{2}\;+a_{5}\times {\left[SO_{2}\right]}_{2}+a_{6}\times {\left[NO_{2}\right]}_{2}+b_{1}\times \left[TS\right]+b_{2}\times \left[U\right]+b_{3}\;\times \left[V\right]+b_{4}\times \left[RH\right]+c_{1}\times \left\{PM_{2.5}\right\}+c_{2}\times \left\{PM_{10}\right\}+c_{3}\;\times \left\{SO_{2}\right\}+c_{4}\times \left\{NO_{2}\right\}+c_{5}\times \left\{O_{3}\right\}$$

where:

(1) PC is the forecast value of the pollutant concentration.
(2) “[]” represents the monitoring values on the initial forecasting day (1 represents the average concentration of the whole area, and 2 represents the average concentration of a single observation site) and the monitoring values of the meteorological element.
(3) “{}” represents the CMAQ products for the forecast data.
(4) *a*_1_L *a_n_, b*_1_L *b_m_, c*_1_L *c_l_* are coefficients and can be calculated by mathematical methods.

**Remark** **13.** *The forecast accuracy of air quality models is largely dependent on the accuracy of the pollution sources and meteorological conditions. Therefore, it is more suitable for short-term pollution forecasting.*

Moreover, the box model, Gaussian model, and K model, as the commonly used air quality models for air pollution forecasting, have some shortcomings. For example, the assumption of the box model is a deviation from the facts, so the concentration forecast value of the boundary layer of the Earth is lower. The Gaussian model has the advantages of being simple and practical and having high spatial resolution, but it has the following deficiencies [89]:

(1) When the simulation scale is up to tens of kilometers or because of an uneven surface of the underlying surface, the flow field is more complex, and it is difficult to meet the requirements of the accuracy of the Gaussian smoke flow model.
(2) Deposition and chemical transformation of the Gaussian model can only be treated roughly, when these processes are very important for the study and the Gaussian model is not applicable.

Moreover, because the K model is derived from the assumption that the gradient transport of molecular diffusion is modeled, it has some limitations:

(1) It is assumed that the gradient transport is required to satisfy certain scale conditions so that the diffusion equation is correct when the smoke flow scale is larger than the dominant eddy.
(2) In the convection condition, the relationship between gradient and transport is not established, so the K model cannot be applied.
(3) The requirements for the basic information and input parameters of K model are very high.

#### 6.1.4. Atmospheric Pollution Forecasts in China

The Hawaii Regional Climate Model (HRCM) model system, which uses an Euler model, was developed by the Institute of Atmospheric Physics of the Chinese Academy of Sciences. The system is composed of the following parts [70]:

(1) Mesoscale meteorological model.
(2) Planetary boundary layer turbulence statistics parameterization (PBLM).
(3) Pollution source model (SM).
(4) Dry and wet deposition model (DSDM).
(5) Concentration calculation model (HRCM).

Its flow chart is shown in Figure 6.

Among them, the (1)–(4) provide the input parameters and the initial field and boundary conditions. The HRCM model is the core of this system, which satisfies the following equations [70]:

(30) $$\frac{\partial c}{\partial t}=-\frac{\partial \left(\mathrm{uc}\right)}{\partial x}-\frac{\partial \left(\mathrm{vc}\right)}{\partial y}-\frac{\partial \left(\mathrm{wc}\right)}{\partial \sigma }+\frac{\partial }{\partial x}\left(k_{x}\frac{\partial c}{\partial x}\right)+\frac{\partial }{\partial y}\left(k_{y}\frac{\partial c}{\partial y}\right)\;+{\left(\frac{g}{P_{L}}\right)}^{2}\frac{\partial }{\partial \sigma }\left(\rho^{2}k_{x}\frac{\partial c}{\partial \sigma }\right)+P_{c}-L_{c}+W_{t}+D_{y}+E_{s}$$

where:

*c = c_j_P_L_*, *c_j_* is the volume mixing ratio of chemical substances, *P_L_ = P_S_* − *P_t_*.

*σ = P* − *P_z_/P_S_* − *P_t_*, *P* is air pressure, *P_t_* is the top pressure of model (*P_t_* = 100 hPa); *P_S_* is the pressure of surface.

$w=\frac{d\sigma }{dt}$ is vertical velocity of *σ* coordinate system.

*P_c_* and *L_c_* are the production and consumption rate caused by the chemical reaction.

*W_t_* is the rate of change in the concentration of material caused by cloud.

*D_y_* is the rate of change of concentration caused by dry deposition.

*E_s_* is a source of pollution.

The required data are the hourly three-dimensional wind field, temperature field, moisture field, turbulent diffusion field, underlying surfaces, hourly ground rainfall, and pollution emission inventory.

The Monte-Carlo multi-source model system is a multi-source Lagrangian model, including transport, diffusion, migration, and transformation processes. This forecast system is similar to the HRCM system, including a mesoscale meteorological model, dry and wet deposition model (DSDM), the planetary boundary layer model (PBLM), and HRCM (Monte-Carlo multi-source model system) [70].

The Monte-Carlo multisource model is developed from a statistical point of view, and the results of the trajectory tracking derive the probability density distribution function *P*:

(31) $$P={\left(kd_{xr}d_{yr}d_{zr}\right)}^{-1}\overset{k}{\underset{j=1}{\sum }}\delta \left[r-r_{j}\left(t-t_{0_{j}}\right)\right]$$

where *r* is the coordinate of the *d_xr_,d_yr_,d_zr_* small volume center at *t* time, and *t*_0*j*_ is the time when a particle *j* is away from the source.

Therefore, the average concentration of pollutants is calculated as follows:

(32) $$c\left(r,t\right)=Q\int_{0}^{t}P\left(t,t|r_{s},t\right)dt$$

The Monte-Carlo multi-source model needs input parameters of temporal and spatial scope, the time and space step, the three dimensional wind temperature field forecasted by the mesoscale-*β* meteorological model in the meteorological field, the temperature with changes in altitude, PBL turbulence statistical parameters, temporal and spatial precipitation distribution, underlying surface types, and sources of pollution data [70].

The CAPPS Model System uses the atmospheric advection diffusion model grid box and never considers the advection diffusion equation chemical reaction of atmospheric pollutants departure [70]:

(33) $$\frac{\partial c}{\partial t}+v\cdot \bar{V}c=\sum q_{i}\delta \left(r_{i}\right)-\bar{V}\cdot \left({\mathrm{cv}}_{d}\right)-\bar{V}\cdot \left({\mathrm{cv}}_{w}\right)+\bar{V}\cdot \overset{=}{k}\cdot \bar{V}c$$

where *c* is the concentration of air pollutants; $\overset{=}{k}$ is the turbulent exchange system; *ν_d_* is the dry deposition velocity; *ν_w_* is the wet deposition velocity; $\sum q_{i}\delta \left(r_{i}\right)$ is in the volume *τ*; and the strength of several sources are located in *r* = (*x_i_*,*y_i_*,*z_i_*) as the sum of the *q_i_* sources.

Integrated within the *τ* range and then volume averaged, the forecast equation of the average concentration in the box is obtained [70]:

(34) $$\tau \frac{\partial \bar{c}}{\partial t}=Q-\oint \int c\left(v+v_{t}+v_{d}+v_{w}\right)ds$$

According to the definition of the pollution index and potential pollution index integral to the above equation, the corresponding expression can be launched. The flow charts are shown in Figure 6.

### 6.2. Meteorological Models

Meteorological models, being part of the air quality model, are commonly used to provide meteorological parameters for air quality models. Or they coupled with other model to simulate the diffusion and trajectories of pollutants. In short, they are rarely used alone. The meteorological models calculate the weather data for the prediction by inputting specific data.

#### 6.2.1. CALMAT Model

CALMET provides three-dimensional meteorological field for the CALPUFF diffusion model, including the diagnostic wind field module and the micrometeorological module. The diagnostic wind farm module generates the first wind field by adjusting the topographic dynamics, slope flow, and terrain obstruction effects for the initial guessed wind field, input the observe data and generate the final wind field through interpolation, smoothing, vertical velocity calculation and divergence minimization. The micrometeorological module uses the surface heat flux, boundary layer height, friction velocity, convection velocity, and other parameters to describe the boundary layer structure based on the parameterized method.

Cartellea et al. established a PrOlor system to forecast environment odor. This system was based on WRF, CALMET and CALPUFF model. Among them, CALMET produced very high resolution meteorological fields over the study domain [90].

#### 6.2.2. WRF and MM5 Model

MM5 is the fifth generation of the National Center of Atmospheric Research/Penn State mesoscale model, WRF is Weather Research and Forecasting model. MM5 and WRF provide the meteorological input fields for vary air quality models, moreover, most studies coupled WRF with chemistry model to simulated and forecast concentration of pollutants (most details, see Section 6.3). In principle, MM5 and WRF are the same but with different characteristics. The simulation results of MM5 and WRF modes were compared by Cheng et al. [91]. The comparisons are shown in Table 14. 

The simulation results show that both MM5 and WRF can simulate the high and low areas of the temperature, but the temperature information forecast by WRF is more consistent with the measured data, and the simulated values of MM 5 are lower than measured values. Comparatively speaking, the WRF simulation of the high and low pressure center position and intensity is closer to the measured pressure field, and the forecast value from MM5 is bigger than the measured pressure. The velocity vector field of MM5 and WRF are consistent with the measured wind field, higher than the measured value. 

In summary, MM5 simulates the high and low value area distribution of temperature, and the pressure and wind field meteorological elements are different from the measured values. Its simulation is worse than the WRF simulation; the relative humidity simulation results of MM5 and WRF are relatively higher than the actual data [91].

### 6.3. Chemical Models

In the real atmosphere, chemical and physical processes affect each other. For example, aerosols can affect the balance of atmospheric radiation. Cloud condensation nuclei can also be formed in the cloud, further affecting precipitation. Weather phenomena such as precipitation, wind, or turbulence can affect the Chemical transport and sedimentation process [92]. So, the chemical model is often used in couple with other models. The most popular chemistry coupling model is Online Coupled Chemistry with WRF. In the coupled model, the air quality component of the model is fully consistent with the meteorological component; such as, the same transport scheme (mass and scalar preserving), the same grid (horizontal and vertical components), and the same physics schemes for subgrid-scale transport [92]. Chuang et al. used WRF/Chem-MADRID to forecast real-time air quality. WRF/Chem-MADRID represents the WRF model with Chemistry combined with the Model of Aerosol Dynamics, Reaction, Ionization, and Dissolution [93]. But the forecast result is not satisfying, the concentration of O_3_ is over-prediction and the concentration of PM_2.5_ is under-prediction, the authors proposed the improvement scheme in the paper from meteorological perspective. Werner et al. applied the on-line WRF-Chem model to forecast the concentration of PM_10_ over Poland. Based on forecast results, the author indicated that WRF-Chem performed better in O_3_ forecast, confirming the significance of the non-linear processes taken into account in an online coupled Eulerian model, but WRF-Chem was difficult to capture the peak, it needs higher resolution sector based emission data and temporal emission profile. [94]. Table 15 lists the main recent studies on the three dimensional models in different urban areas.

**Remark** **14.** *In recent years, the air quality simulation technology has developed rapidly, in particular, a model that combines geographic information and meteorological data. Currently, various air quality models have been widely used in environmental impact assessment, major scientific research, and environmental management and decision making, but they also encountered many problems in the practical application. Although simulation results of complex advanced model are good, the heavy calculation burden makes it infeasible in practical application. Therefore, finding an alternative model or simulating simplification is still an important problem.*

## 7. Hybrid Systems

A hybrid system (HS) is characterized by a combination of any two or more of the methods [96]. The purpose of the HS model is to utilize the advantages of each method and improve the accuracy of forecasting as much as possible. 

### 7.1. PCA-ANN

Mishra et al. proposed a hybrid model that combined statistical regression with a specific computational intelligence method for forecasting hourly NO_2_ concentrations at the Taj Mahal in Agra, India [25]. At first, they used PCA to find the correlations between meteorological forecasting variables and air pollutants. Then, the significant variables were taken as the input parameters to propose the reliable physical ANN–multi layer perceptron model for forecasting air pollution in Agra. The forecast results are given in Table 16.

The result indicated that the ANN-MLP model could not forecast well during high concentration pollution periods. However, the anthropogenic activities are the most important variables for forecasting. 

### 7.2. Multilayer Perceptron Neural Network and Clustering Algorithm

Clustering analysis, also called group analysis, is a kind of multivariate statistical analysis method for the classification of samples or indicators. The object of discussion is a large number of samples, which can be reasonably classified according to their respective characteristics; this classification does not need prior knowledge or a model, and it can be used as reference.

*K*-*Means algorithm*. *K*-means is one of the unsupervised learning algorithms that solve clustering problems. *K*-means clustering is a clustering method used for a given cluster number *k*. The main idea is to randomly select *K* objects as the initial cluster centers, then calculate the distance between each object and the cluster center and assign each object to the nearest cluster center. This algorithm aims at minimizing the squared error function of the objective function. The objective function is [60]:

(35) $$J=\overset{k}{\underset{j=1}{\sum }}\overset{n}{\underset{i=1}{\sum }}\|x_{i}^{\left(i\right)}-c_{j}{\|}^{2}$$

where $\|x_{i}^{\left(i\right)}-c_{j}{\|}^{2}$ is a chosen distance measured between a data point *x_i_*^(*i*)^ and the cluster *c_j_*. This is an indicator of the distance of the n data points from their cluster centers [60].

*Fuzzy c–Means (FCM) algorithm.* This clustering method allows one piece of data to belong to two or more clusters, and each element is associated with a set of membership levels. The algorithm is based on optimizing the objective function given by Equation (40) [60]:

(36) $$J_{FCM}\left(Z,U,V\right)=\overset{c}{\underset{i=1}{\sum }}\overset{N}{\underset{k=1}{\sum }}\left(\mu_{ik}\right)\|z_{k}-v_{i}{\|}^{2}$$

where the matrix U = [*μ_j_*]ò*M_FCM_* is a fuzzy partition of the data set *Z*, and *V* = [*ν*_1_,*ν*_2_,L_,_*ν*_c_] is the vector of prototypes of the clusters, which are calculated according to $D_{ikA}=z_{k}-\nu_{i}^{2}.$ This is a square inner product distance norm. The optimal partition U* of *Z* for the FCM algorithm is reached through the couple (U*,V*) that minimizes locally the objective function *J_FCM_* according to the alternating optimization.

Cortina-Januchs et al. [60] implemented clustering algorithms (*K*—means and FCM) to build the patterns as follows:

(37) $$P=\left[C_{PM_{10}},WS,WDI,T,HR\right]$$

(38) $$WDI=1+sin\left(WD+\frac{\pi }{4}\right)$$

where, $C_{PM_{10}}$ is the *PM*_10_ concentration; *WS* represents wind speed; *WDI* is the Wind Direction Index (WDI); *T* is temperature; *HR* is the relative humidity. 

The best forecast results for the three stations are shown in Table 17. The time window indicates the number of hours needed to make the forecast.

Authors indicated that clustering algorithms can add useful information to the ANN by identifying groups with similar data characteristics and finding relationships between them that would not be obtained from other methods.

### 7.3. Hybrid Artificial Neural Network and Hybrid Support Vector Machine

Grivas et al. developed a model that uses a combination of meteorological data and time-scales as input variables for the ANN [22]. There are four methods in this study: feed forward multi-layer perceptron (FFMLP) NN, multi-layer perceptron (MLP) based on a genetic algorithm (GA) optimization procedure (mainly used to select input variables), MLP developed without meteorological input variables, and MLR. Finally, they compared the forecasting ability of these models. The results can be seen in Table 18.

The differences in Table 18 indicate that FFMLP and GA-MLP are more effective than MLPnomet and MLR. In other words, the forecasting model will work better if more elements are considered or a hybrid model is proposed and applied. 

### 7.4. CS-EEMD-BPANN Model

Qin et al. proposed the CS-EEMD-BPANN model for forecasting PM concentrations. This hybrid method is based on grey correlation analysis (GCA), ensemble empirical mode decomposition (EEMD), Cuckoo search (CS), and BP artificial NNs (BPANN). The steps to build the model are as follows [97]:

***Step 1.*** Selection of appropriate predictors based on gray correlation analysis. Some air pollutants (CO, NO_2_, O_3_, and SO_2_) and meteorological factors (WS/D, T, H, and P) might affect the PM concentration, and using the gray correlation analysis to obtain the influence law of PM is a primary concern.

***Step 2.*** Use the EEMD technique to filter out the white noise or useless information for selecting influencing factors and PM concentration.

***Step 3.*** The data sets with the noise removed are input into the BPANN model to obtain the forecasted values. In this study, the forecast model is a novel BPANN-based multi-step-ahead forecasting model, and the CS algorithm is used to optimize the connection weights and thresholds of the BPANN architecture to make it more stable.

The authors used data that were measured in the winter of 2013–2014 in Beijing, Shanghai, Guangzhou, and Lanzhou, and the results are shown in Table 19.

It can be found from the comparisons that the CS-EEMD-BPANN model performs better.

### 7.5. ICEEMD-SVM-WOA

Xu et al. proposed a hybrid air quality early-warning system, which is combined with ICEEMD, SVM, and WOA. The steps of the hybrid method can be summarized as follows [52]:

***Step 1.*** ICEEMD is used to decompose the original time series into several intrinsic mode functions (IMFs) for eliminating the negative influence of noise and to exploring the inner characteristics of the data Compared with CEEMD model, the ICEEMD model is mainly improved from two aspects: (a) CEEMD modes contain some residual noise; (b) the signal information appears “later” than in EEMD with some “spurious” modes in the early stages of the decomposition [98].

***Step 2.*** The SVM optimized by WOA is employed to build a predictor for each IMF. SVM is used to predict each IMF, among them, WOA is used to obtain the proper weight coefficient of each predictor. The leave-one-out strategy is performed to integrate all forecasted IMFs and then obtain the final forecast result.

The ICEEMD-SVM-WOA model results in study areas are shown in Table 20.

It’s proved that the hybrid model ICEEMD-SVM-WOA is superior to the other four benchmark models used in this study. To facilitate the comparison, the above mentioned hybrid models are summarized in Table 21.

**Remark** **15.** *With the popularity of hybrid systems, more and more scholars construct air quality forecasting system based on the HS. Generally, air quality forecasting system contains three modules: data pre-processing module, optimization module, and forecasting module. The function of data pre-processing is eliminate chaotic noise and extract effective features that lie in original series; optimization module aims to optimize the parameters utilized in the forecasting module to improve the forecasting accuracy. The research process for an air quality forecasting system is usually divided into three steps.*

***Step 1.*** *Decompose the original series. Some signal processing tools are used in this step, such as wavelet transform, short-time Fourier transform, and EEMD. The main purpose of signal processing is to weaken the redundant content in the signal, remove the mixed noise and interference, and transform the signal into a form for easy processing and analysis for subsequent research.*

***Step 2.*** *Optimize the forecast model. There are many methods available in this step, such as genetic optimization algorithms, Ant colony optimization algorithms, and whale optimization algorithms.*

***Step 3.*** *Construct the forecast model. This is an important step in the study, and several methods can be chosen, such as statistical methods (regression, principal component analysis, etc.) and AI methods (ANN, wavelet NN, etc.).*

*When constructing a hybrid model, we should take into account the specific situation of the study areas to choose different models for signal decomposition, forecasting, and optimization. Finally, the hybrid model is used for predictions.*

## 8. Other Methods of Air Pollution Forecasting 

In addition to the common models described above, some scholars forecast by using new models. The most popular method is the hybrid system, and more and more scholars forecast air pollution by applying hybrid models and indicate that the accuracy of the hybrid model is higher than individual models. Moreover, some researchers use common models to forecast, taking into account geographic factors. This section will describe these models in detail. 

### 8.1. Geographic Methods

Kurt et al. believed that using geographical factors in the experimental area for air pollution forecast can improve the accuracy of forecasting [26]. Therefore, when forecasting the air pollution, the pollution situation in the nearby area can be used as a reference. The interaction between pollutants is determined by the different geographic terrain, the geometric characteristics of the building, and other geographical and environmental attributes. These complex interactions play a significant role in the forecasting of air pollution. In addition, the location and distances between districts are also important, generally, the closer the distance is, the higher the similarity is. [26]. So, Kurt et al. presented three geographic models with the increasing order of complexity to forecast SO_2_, PM_10_ and CO concentration in Istanbul, these methods are described in Table 22.

**Remark** **16.** *Few forecasts considered the geographical factors of neighboring cities. However, Kurt et al.* [26] *proved that the error in geographic methods was always lower than that in a non-geographic model. The best neighborhood and the minimum error produced on an experimental date can be determined experimentally. However, there are some drawbacks for geographic models; for example, it is difficult to choose the proper neighboring districts and models because the error may be higher between two cities in a single-site neighborhood model but lower in other models, so it needs many comparative analyses.*

### 8.2. Grey System (GM)

Grey system theory was initiated by Deng in 1982, mainly in the case of uncertainty and lack of information, making full use of historical data to build a model. Gray system prediction model is expressed by differential equation [62], generally, the grey model (GM) is written as GM (*m*, *n*), and in which m is the order of the differential equations and n is the number of variables of the model. Thus, GM (1, 1) is short for “grey model first order one variable,” which is the dominant model of the grey forecasting theory in grey systems theory. GM (1, 1) has been widely used in forecasting studies because of its advantages, low requirement for data items to build forecasting models and higher forecast accuracy as compared with other forecasting methods [6]. 

There are four kinds of commonly used gray forecasting models:

(1) Sequence forecast: A grey forecast model that can reflect the characteristic of the forecast object is constructed based on the observation of the time series.
(2) Catastrophe and abnormal value forecast: Using a grey model to forecast the time that the abnormal value appears and the time that the abnormal value appears in the specific time zone.
(3) Topology forecast: Using the original data curve and finding all the time points in which the fixed value occurs on the curve. The fixed value is used as the frame structure and the number of time points. The model is established to forecast the time point of the fixed value.
(4) System forecast: Establishing a set of interrelated grey forecasting models for the system behavior characteristic and forecasting the change of the coordination among numerous variables in the system.

Pan et al. employed a grey dynamic model group and grey relational analysis to forecast the air quality change trend of Tianjin, and the forecasting result indicated that model group had high accuracy [103]. An-order multiple grey system (GM (1, N)) optimized by grey-genetic algorithm was proposed by Tsai et al., who used this novel model to forecast the air pollution in Taiwan. The result indicated that the grey-genetic algorithm can refine the prediction accuracy of GM models [104]. At first, the nonlinear prediction model GM (1, 1) is established because the relationship between the variables in the atmospheric environment system is nonlinear. The data sequence is then cumulatively generated, and the forecasted values are obtained by correlation calculation. Finally, the model is diagnosed and the reliability of the model is analyzed. If the test statistic is within the allowable range, the predicted value can be calculated; otherwise, it is necessary to modify it by analyzing the residual sequence and then making a prediction.

**Remark** **17.** *In practical application, it is found that when using a GM prediction model to predict, sometimes good prediction results can be obtained, but sometimes the prediction results are not accurate. Scholars indicate that this is because those researchers ignore the premise of using GM prediction: the original data sequence must satisfy the exponential law, and the speed of data sequence change must be slow. Moreover, GM prediction models that have an inherent unavoidable error because this biased index model is a small sample prediction method, and the precision depends on the conformation of the background value and the selection of original condition.*

### 8.3. Natural Source Pollution Forecasting

In addition to man-made air pollution, natural sources must not be ignored. Wildfires, such as forest fire and agricultural burning, always produce heavy smoke, which is harmful to human respiratory system. The volcanic ash contains not only water-insoluble particles matter, but also heavy metal, and that are harmful to the human body, meanwhile, affecting human life and productive activities. If the transport and dispersion processes of wildfires and volcanic ash can be simulated, adverse effects and losses can be reduced. To the best of our knowledge, the development and the spread of pollutants are more dependent on the weather, so most of fire smoke and volcanic ash disperses models are based on weather forecast [95].

Bhoi et al. use the case of forest fire in the Eastern United States to forecast the emission of PM_2.5_ and CO in wildfires. In their proposed framework, Operational Multiscale Environment modeled with Grid Adaptivity, Real-time remote sending data were used to automatically detect fire pixels, and the output was generated in GIS format. This system will help to assign persons involved in wildfires management, improved work efficiency and reduce fire damage [105].

Kochanski et al. coupled an atmosphere-fire model named WRF-SFIR and WRF-Chem to simulation and forecast the smoke emission and dispersion. First of all, WRF-SFIRE conversion the standard fuel categories to the Moderate Resolution Imaging Spectroradiometer (MODIS) land cover types, and after that, the fuel consumption rates for each fire grid based on the mas of fuel burnt in one time step was calculated. Next, the emission fluxes were calculated as the products of the consumption rates and the fuel-specific emission factors. Finally, the computed results described above were put into the WRF-Chem to obtain simulation and forecasting result. This model coupled the atmosphere-fire and atmosphere-chemistry in high level, giving an opportunity for studying complex interactions between the fire and the atmosphere [95]. 

Another popular framework is BlueSky, and it is a smoke model to simulate the cumulative smoke impacts from fires [106]. This model includes input module and output module, and the fire information and meteorological information as the initial input data are entered into the model primarily, meanwhile fuel loadings and moisture conditions are determined, and consumption is calculated. The emission from the consumption are speciated and allocated diurnally, then the dispersion and trajectory models were drive by these emissions. In this model, WRF and MM5 are used to provide meteorological parameter, CALPUFF is applied to simulate the dispersion and trajectory, and CALMET is employed to provide meteorological field for the CALPUFF [106]. 

Goodrick et al. concluded the smoke transport model. They indicated that models for forecasting the effects of wildfire smoke consisted of four basic components. The first part included the description of the emission sources such as pollutants and heat release. The second part determined the vertical range of the plume by checking the stability of the atmosphere, the wind profile and the rate of the exothermic fire source. The third part was the actual smog movement (transportation and diffusion) of environmental winds. The fourth part explored the chemical transformation of smoke constituents to explore a series of air quality issues [107]. They also introduced several prediction models, for more details please see [107].

To facilitate the comparison, we summarize studies regarding different methods in Table 23.

**Remark** **18.** *From Table 23, it can be observed that many approaches have been developed for air pollution forecasting, and each method has its own characteristics. In addition, the specific problems are that the pollutants are different and the specific factors in the models are quite different, so it is difficult to select the most suitable approach for forecasting air pollution. Researches should not only consider the advantages of forecasts but also the disadvantages.*

*Statistical models require a large amount of historical data and have a high dependence on data time series. AI methods are unstable and have a high dependence on data. The process of building hybrid models is a little complex. Therefore, it is necessary to make a full comparison of these methods and determine the most appropriate method for forecasting.*

## 9. Conclusions

As a serious concern, air pollution in the 21st century has received great attention in recent years, and various air pollution forecasting methodologies and approaches have been advanced. This work mainly reviewed the methods of air pollution forecasting. At the beginning of this work, we reviewed the current research status of air pollution from the perspective of pollution emission inventories, health effects, and air pollution assessment to air pollution control efficiency and air pollution early warning systems. Then, we reviewed the methods of air pollution forecasting as the core of this work. Based on relevant literature, these methods can be roughly divided into three categories: potential forecast model, three dimensional forecast methods, and hybrid system. These methods have advantages and disadvantages. According to the application of these methods in the forecast, the conclusions of these methods and models are summarized below:

- Statistical models have a wide application and require less time to build models, but they require a large amount of historical data and have a high dependence on the data time series approach.
- AI methods, such as the NN approach, have good performance and can solve nonlinear data, but the models are unstable and have a high dependence on data. Moreover, most optimization algorithms are easy to be understood and combined with other methods; however, they easily fall into local optima.
- As the most popular method, a hybrid system has good robustness with low risk and strong adaptability and can take advantage of other models. However, the process of building models is relatively complex.
- Traditional AI performance is better than that of statistical methods, but worse than that of the hybrid model.
- Processed original series did better than the unprocessed original series in terms of air pollution forecasting.
- It is proven that forecast performance is better when considering the meteorological variables and the geographic factors.

In conclusion, as the atmospheric environment is a complex system, there are many factors affecting the quality of the atmospheric environment, and the relationship between them is complicated. Therefore, air pollution forecasting based on the area and different pollutants should choose different forecasting methods. Moreover, there is no one best approach to make the most accurate forecast.

## Figures and Tables

**Table 1:** List of assessment methods.

List of Assessment Methods		
Types	Main Equations	Meaning of Variables
Market value method	$S_{1}=\sum_{i=1}^{n}P_{i}\times \Delta R$	*S*_1_ is the economic loss of environment quality; *P* is the market price of good *i*; Δ*R* is the yield reduction of good *i* that caused by pollution and ecological damage.
Opportunity cost method	$S_{2}=V_{2}\times W_{2}$	*S*_2_ is the opportunity cost of the loss; *V*_2_ is the Unit opportunity cost of the certain resource; *W*_2_ is the amount of resources being polluted or damaged.
Engineering cost method	$S_{3}=V_{3}\times Q$	*S*_3_ is the cost of prevention and controlling pollution or destruction; *V*_3_ is the unit costs of protecting, restoring or replacing the original environmental functions; *Q* is the unit costs of protecting, restoring or replacing the original environmental functions;

**Table 2:** Nomenclature of methods.

Abbreviation	Explanation	Abbreviation	Explanation
ADMS	Atmospheric Dispersion Modelling System	GM	Gray model
AI	Artificial intelligence	GCA	Gray correlation analysis
ANN	Artificial neural network	GRNN	General regression neural networks
ANF	Adaptive neuro-fuzzy	HF	Hybrid forecast
ARIMA	Autoregressive integrated moving average	HS	Hybrid system
ANFIS	Adaptive neural network fuzzy inference system	ICEEMD	Improved complementary ensemble empirical mode decomposition
BPNN	Back-propagation neutral networks	KF	Kalman filter
CAMx	Comprehensive Air Quality Model with Extensions	MLP	Multi-layer Perceptron
CALPUFF	California Puff model	MLR	Multiple-linear regress
CALMET	California Meteorological Model	MM5	Mesoscale Model 5
CS	Cuckoo search	PCR	Principal component regress
CMAQ	Community Multi-scale Air Quality	PCA	Principal component analysis
CEEMD	Complete ensemble empirical mode decomposition	PP	Projection pursuit model
CERC	Cambridge Environment Research Corporation	RM	Rolling mechanism
DEA	Data Envelopment Analysis	SVM	Support vector machine
EMD	Empirical mode decomposition	SVR	Support vector regression
EEMD	Ensemble empirical model decomposition	SWT	Stationary wavelet transform
FCM	Fuzzy c–Means algorithm	SSA	Singular spectrum analysis
FTS	Fuzzy time series	WOA	Whale optimization algorithm
FFNN	Feed-forward neural networks	WRF	Weather Research and Forecasting Model
FFMLP	Feed forward multi-layer perception	WRF-Chem	Weather Research and Forecasting Model coupled with Chemistry
GA	Genetic algorithm		

**Table 3:** The definitions and formulas of indexes involved in this paper.

Metric	Definition	Equation
MAE	The mean absolute error of *N* forecasting results	$MAE=\frac{1}{N}\overset{N}{\underset{i=1}{\sum }}\left|F_{i}-A_{i}\right|$
MSE	The mean squared error of *N* forecasting results	$MSE=\frac{1}{n}\overset{n}{\underset{i=1}{\sum }}{\left(F_{i}-A_{i}\right)}^{2}$
RMSE	The square root of average of the error squares	$RMSE=\sqrt{\frac{1}{N}\times \overset{N}{\underset{i=1}{\sum }}{\left(F_{i}-A_{i}\right)}^{2}}$
NMSE	The normalized average of the squares of the errors	$NMSE=\;\frac{1}{N}\overset{N}{\underset{i=1}{\sum }}\frac{{\left(F_{i}-A_{i}\right)}^{2}}{F_{i}A_{i}}$
MAPE	The average of *N* absolute percentage error	$MAPE=\;\frac{1}{N}\overset{N}{\underset{i=1}{\sum }}\left|\frac{A_{i}-F_{i}}{A_{i}}\right|\times 100\%$
IA	The index of agreement of forecasting results	$IA=\;1-\overset{N}{\underset{i=1}{\sum }}{\left(F_{i}-A_{i}\right)}^{2}/\overset{N}{\underset{i=1}{\sum }}{\left(\left|F_{i}-Ā\right|+\left|A_{i}+Ā\right|\right)}^{2}$
R	The correlation coefficient	$R=\;\frac{\left(A_{i}-Ā\right)\left(F_{i}-\bar{F}\right)}{\sigma_{F}\sigma_{A}}$
AE	The absolute error of forecasting results	$AE=\;\left|F_{i}-A_{i}\right|$
FB	The fractional bias of N forecasting results	$FB=\;2\left(Ā-\bar{F}\right)/\left(Ā+\bar{F}\right)$
IOA	The index of agreement	$IOA=1-\frac{\sum_{i=1}^{N}{\left(F_{i}-A_{i}\right)}^{2}}{\sum_{i=1}^{N}{\left(\left|F_{i}-\bar{F}\right|+\left|A_{i}+Ā\right|\right)}^{2}}$

**Table 4:** Transformation of the nonlinear regression and linear regression.

Types	Nonlinear Function	Do Transformation	Linear Function
Hyperbolic function	$Y=a+b\frac{1}{x}$	$x'=\frac{1}{x}$	Y'=a+bx'
Power function	$Y=ax^{b}$	$Y^{\prime }=\ln Y\;\;x=\ln x\;\;A=\ln a$	Y'=A+bx'
Exponential function	$Y=ae^{bx}$ or $Y=ae^{\frac{b}{x}}$	$Y^{\prime }=\ln Y,\;A=\ln a$ or $Y^{\prime }=\ln Y,\;x=\frac{1}{x}\;,\;A=\ln a$	Y'=A+bx' or Y'=A+bx'
Logarithmic function	Y=a+blnx	x'=lnx	Y'=a+bx'
S curve type	$Y=\frac{1}{a+be^{-x}}$	$Y^{\prime }=\frac{1}{Y},\;\;x=e^{-x}$	Y'=a+bx'
Parabolic type	$Y=a+bx+cx^{2}$	$x_{1}=x,x_{2}=x^{2}$	$Y^{\prime }=a+bx_{1}+cx_{2}$

**Table 5:** Forecast accuracy of possible SARIMA model.

SARIMA	MAPE	MAE	MSE	RMSE
**Pasir Gudang**				
(0,1,1)(0,1,1)^12^	11.08	5.39	37.76	6.14
(0,1,1)(1,1,0)^12^	11.08	5.77	44.50	6.67
**Johor Bahru**				
(1,1,0)(1,1,0)^12^	15.28	7.06	76.05	8.72
(1,1,0)(0,1,1)^12^	9.99	4.12	21.90	4.68
(0,1,1)(1,1,0)^12^	19.13	8.87	120.69	10.99
(0,1,1)(0,1,1)^12^	9.77	4.22	23.82	4.88
**Muar**				
(1,1,0)(0,1,1)^12^	12.20	5.42	49.13	7.10
(0,1,1)(2,1,0)^12^	11.32	5.10	38.62	6.21
(0,1,1)(0,1,1)^12^	10.44	4.84	33.49	5.79

**Table 6:** Division of $C_{i}^{1}$ values.

Grade	1	2	3	4	5
Range of $C_{i}^{1}$ values	(0 ≤ $C_{i}^{1}$ ≤ 0.2)	(0.2 ≤ $C_{i}^{1}$ ≤ 0.4)	(0.4 ≤ $C_{i}^{1}$ ≤ 0.6)	(0.6 ≤ $C_{i}^{1}$ ≤ 0.8)	(0.8 ≤ $C_{i}^{1}$ ≤ 1)

**Table 7:** PP regression forecast result.

Actual Type	Forecast Type	Absolute Error	Relative Error
2	2.399	0.399	19.9%
3	5.632	2.632	87.7%
4	4.298	0.298	7.5%
5	5.439	0.439	8.8%

**Table 8:** Forecast accuracy of ANN of pollutants.

Pollutants	Station 1		Station 2		Station 3		Station 4	
	MAE	RMSE	MAE	RMSE	MAE	RMSE	MAE	RMSE
SO_2_	0.0674	0.0910	0.0524	0.0929	0.0386	0.0636	0.0512	0.0870
PM_10_	0.0428	0.0631	0.0476	0.0615	0.0485	0.0740	0.0494	0.0872

**Table 9:** Comparisons result with different forecasting methods.

Study Areas	Methods	MAE	MSE	RMSE
Pasir Gudang	SARMIA	5.39	37.76	6.14
	FTS	5.88	53.43	7.31
	ANN	3.87	32.09	5.66
Johor Bahru	SARMIA	4.12	21.90	4.68
	FTS	5.21	33.82	5.82
	ANN	2.70	12.79	3.58
Muar	SARMIA	4.84	33.49	5.79
	FTS	3.49	18.44	4.29
	ANN	3.29	18.05	4.25

**Table 10:** Comparison of the forecasting performances using different models.

Model	Air Pollutants	Performance Criteria	
		MAPE	RMSE
W-BPNN	PM_10_	15.277	15.391
	SO_2_	15.886	8.269
	NO_2_	16.544	2.621
BPNN	PM_10_	31.266	23.624
	SO_2_	22.119	12.716
	NO_2_	35.030	5.406

**Table 11:** Short summary of commonly used wavelet.

Wavelet	Main Equations	Description
Haar wavelet	$\psi_{H}\{\begin{array}{c} 1,0\leq x\leq \frac{1}{2}\\ -1,\frac{1}{2}\leq x\leq 1\\ 0,\;Others \end{array}$	Haar function is the earliest use of wavelet analysis in the wavelet, and is also the simplest wavelet. The function itself is a step function
Mexican Hat wavelet	$\psi \left(x\right)=\frac{2}{\sqrt{3}}\pi^{-\frac{1}{4}}\left(1-x^{2}\right)e^{-\frac{x^{2}}{2}}$	Mexican Hat wavelet is the two-order derivative of Gauss function (plus minus)
Morlet wavelet	$\psi \left(x\right)=ce^{-\frac{x^{2}}{2}}\cos\left(5x\right)$	Morlet wavelet does not have orthogonality and no compact support set, so it can only satisfy the condition of continuous wavelet, but cannot be discrete wavelet transform and orthogonal wavelet transform
Daubechies wavelet	${\left|m_{0}\left(\omega \right)\right|}^{2}={\left(\cos^{2}\frac{\omega }{2}\right)}^{N}P\left(\sin^{2}\frac{\omega }{2}\right)$ $m_{0}\left(\omega \right)=\frac{1}{\sqrt{2}}\sum_{k=0}^{2N-1}h_{k}e^{-jk\omega }$	Assuming, $P\left(y\right)=\sum_{k=0}^{N-1}c_{k}^{N-1+k}y^{k}$ among them, is the binomial coefficient; Daubechies wavelet function is the standard orthogonal wavelet, which makes it possible to analyze the discrete wavelet transform.

**Table 12:** Forecast accuracy of SVM of SO_2_ and PM_10_.

Pollutants	Station 1		Station 2		Station 3		Station 4	
	MAE	RMSE	MAE	RMSE	MAE	RMSE	MAE	RMSE
SO_2_	0.0477	0.0840	0.0491	0.0866	0.0266	0.0498	0.0358	0.0602
PM_10_	0.0393	0.0606	0.0341	0.0518	0.0468	0.0739	0.0420	0.0756

**Table 13:** Forecast accuracy in testing period of FTS.

Study Areas	MAE	MSE	RMSE
Pasir Gudang	5.88	53.43	7.31
Johor Bahru	5.21	33.82	5.82
Muar	3.49	18.44	4.29

**Table 14:** Comparison of MM5 and WRF model.

Project	MM5 Model	WRF Model
Vertical coordinate	Terrain following height coordinates	Terrain following quality coordinates
Conservation	Not necessarily conservative	Conservation of mass, momentum and scalar quantity
Time integral	Leapfrog integration scheme	Three order Runge-Kutta integral scheme
Horizontal convection	Second order accuracy center format	Five order upwind difference scheme
Damping filter	Four order smoothing	No requirement
Typical time step	3 times the distance of the grid	6 times the distance of the grid

**Table 15:** Recent studies on air pollution forecasting using three dimensional model.

Method	Pollutant	Country	Inputs	Ref.
WRF-Chem	PM_10_	Poland	Meteorological data, emission data	[94]
Models-3/CMAQ	O_3_	United States	Meteorological information, emission rates from sources	[27]
CMAQ-MOS	PM_10_, NO_2_	China	Wind field (U, V), temperature field (Ts), relative humidity (RH)	[65]
CMAQ-ANNs	PM_10_, SO_2_	China	Wind field (U, V), temperature field (Ts), relative humidity (RH), concentrations of PM_2.5_, PM_10_, SO_2_, NO_2_, O_3_	[65]
WRF-ADMS	Perfluoromethylcyclohexane	Tunis	Initial and boundary conditions, topography, land use and soil data, exit diameter, release point height, flow rate, temperature, hourly averaged meteorological data	[83]
Coupled WRF-SFIRE with WRF-Chem	Fire somke	United States	Fuel categories, FINN emission factors,	[95]
CALPUFF-WRF	SO_2_	Sultan	land use categories, terrain elevations, surface and upper air meteorological observations or meteorological fields	[85]
WRF-Chem	O_3_	United States	No detailed description	[92]
WRF/Chem-MADRID	O_3_, PM_2.5_	United States	No detailed description	[93]
CALPUFF	Total suspended particulate (TSP)	Israel	Temperature, relative humidity, barometric pressure, 10 min average wind speed and direction, cloud cover, topographic data	[84]
AERMOD	Total suspended particulate (TSP)	Israel	Meteorological data (Temperature, relative humidity, barometric pressure, 10 min average wind speed and direction) from two site, cloud cover, topographic data	[84]

**Table 16:** Statistical performance measures for ANN–MLP model.

Statistical Measures	Ideal Value	Training Value	Validation Value
R	1	0.89	0.91
IOA	1	0.99	0.98
NMSE	0	0.016	0.017
FB	0	0.001	−0.021

**Table 17:** Results for forecast of the average concentration of PM_10_ for the next day.

Stations	Clustering Algorithms	Time Window	Number of Cluster	MAE	MSE
CRUZ ROJA (CR)	*K*–means	1	8	0.0207	0.00085
	FCM	1	7	0.0208	0.00083
Nativitas (NA)	*K*–means	1	2	0.0230	0.00087
	FCM	2	5	0.2031	0.00095
DIF (DF)	*K*–means	3	8	0.0280	0.00134
	FCM	1	3	0.0257	0.00113

**Table 18:** Performance indicators for the developed forecast models.

Stations	Metric	FFMLP	GA-MLP	MLP_nomet_	MLR
Station 1	MAE	14.03	15.36	18.91	17.46
	RMSE	20.28	22.39	27.87	26.68
	R	0.78	0.73	0.53	0.59
	IA	0.87	0.83	0.65	0.72
Station 2	MAE	14.18	14.48	16.99	17.37
	RMSE	19.36	19.26	22.47	23.90
	R	0.70	0.65	0.48	0.53
	IA	0.80	0.79	0.63	0.65
Station 3	MAE	19.08	20.55	27.49	24.53
	RMSE	26.06	28.70	38.11	35.14
	R	0.80	0.73	0.43	0.55
	IA	0.88	0.83	0.56	0.64
Station 4	MAE	7.68	7.54	10.25	11.94
	RMSE	12.35	12.16	16.62	17.06
	R	0.82	0.83	0.54	0.55
	IA	0.89	0.90	0.65	0.65

**Table 19:** The performance of forecast model.

Stations	CS-BPANN		EEMD-BPANN		CS-EEMD-BPANN	
	AE	MAPE	AE	MAPE	AE	MAPE
Station 1	1.71	11.27%	-	-	1.583	9.37%
Station 2	15.45	18.53%	13.82	17.56%	13.86	15.78%
Station 3	28.56	41.04%	28.16	40.59%	27.64	36.98%

GCA is initially used to identify the major factors influencing PM. Gray relational order is examined between the PM and potential factors. Forecasting result is improved by 24%, 16%, 16% and 13% for different strategies. The developed model could be used in sites with different characteristics. Proposed method CS-EEMD-BPANN is more stable than BPANN and EEMD-BPANN.

**Table 20:** Forecast results of ICEEMD-SVM-WOA model in three study areas.

Study Areas	PM_2.5_		PM_10_		SO_2_		NO_2_		CO		O_3_	
	MAE	MAPE	MAE	MAPE	MAE	MAPE	MAE	MAPE	MAE	MAPE	MAE	MAPE
Taiyuan	3.197	9.204	5.517	6.689	1.497	7.831	1.765	5.614	0.024	2.820	3.392	4.225
Harbin	1.781	2.260	3.203	7.457	0.533	9.351	2.420	7.236	0.023	2.921	3.430	7.900
Chongqing	2.900	8.795	5.263	10.311	1.160	13.219	2.882	8.265	0.049	5.005	4.350	11.514

**Table 21:** The short summary of hybrid system for air pollution.

List of Recent Research on the Application of HS in the Field of Air Pollution	
Author	Main Contribution
Chen et al. [17]	Combining numerical forecast (WRF) with statistical analysis (temporal synoptic index) to forecast high-PM_10_ concentration in Beijing. This hybrid forecast system forecasts high-PM pollution events is more accurately than current forecast methods. It combines the strengths of various methods while avoiding the disadvantages found when statistical forecast methods are used alone.
Zhou et al. [99]	Established a hybrid EEMD-GRNN model to forecast the concentration of pollutants in Xi’an, which was shown to be superior to other conventional models.
Qin et al. [97]	Proposed the CS-EEMD-BPANN model for forecasting PM concentrations in Beijing, Shanghai, Guangzhou and Lanzhou. The forecasting result is improved and this method is more stable than BPNN and EEMD-BPANN.
Qin et al. [100]	Using an a priori algorithm mined the spatial and temporal associations of intercity *PM*, also mined cross spatial and temporal associations of *PM*_10_ and *PM*_2.5_ in the Jing-Jin-Ji region (China).
Wang et al. [68]	They used HANN, HSVM and Taylor expansion forecasting model in Taiyuan. The innovation involved in this approach is that it sufficiently and validly utilizes the useful residual information on an incomplete input variable condition.
Feng et al. [101]	1. Using trajectory based geographic parameter as an extra input to ANN model; 2. Applying forecast strategy at different scales and then sum them up; 3. The backward trajectories from Hybrid Single-Particle Lagrangian Integrated Trajectory (HYSPLIT) model were used to track the transport corridors of air masses.
Xu et al. [52]	Proposed ICEEMD-SVM-WOA model and FE model. This model not only forecast the concentrate on air pollutants, but also evaluates the effectiveness of the new forecast system by fuzzy evaluation method.
Wongsathan et al. [102]	Proposed a fundamental hybrid forecast model. This model can improve the performance of the forecast models, the exogenous variable may be considered as well as the modified of the hybrid algorithm

**Table 22:** The description of three geographic models.

Model	Description
Single-site neighborhood model	The main idea of this model is to use the air pollution index of one or more neighboring regions as the input variables of the forecast area.
Two-site neighborhood model	This model considers two neighboring districts. The rationale for this model is that using more predictor variables should achieve higher accuracy.
Distance-based model	In this model, the weighted average value of air pollutants is calculated according to the distance between the adjacent regions and the forecasted distance. The model is based on the idea that the effects of air pollutant levels of the neighboring district are inversely proportional to the distance between the two districts.

**Table 23:** Different models of air pollution forecast.

Method Types	Authors	Models	Main Conclusions
Statistical methods	Silibello et al. [108]	Kalman filter (KF) and Hybrid forecast (HF)	Use two adjustment techniques, the HF and the KF, to improve the accuracy of forecasting supplied by an air quality forecast system
	Huebnerova et al. [109]	Generalized linear models with log–link and gamma distribution	It’s shown that the predicted meteorological variables are used to predict well though comparative analysis of the two models
Artificial intelligence methods	Catalano et al. [110]	ANN and ARIMAX	Forecasted the extreme concentrations by integrating the two models into an ensemble
	Feng et al. [111]	SVM-GABPNN	Proposed a hybrid model which SVM was used to classify data, GA used to optimize the BPNN model.
	Bai et al. [24]	W-BPNN	Using wavelet transform to realize feature extraction and characterization of air pollutants
	Siwek et al. [112]	Wavelet transformation, the multilayer perceptron, radial basis function, Elman network, SVM and linear ARX model	Decomposed the data into the wavelet coefficients and used different NN to individual prediction, then combined the few predictors in the ensemble. This approach does not require very exhaustive information about air pollutants, and it has the ability of allowing the nonlinear relationships between very different predictor variables.
Hybrid methods	Feng et al. [101]	Hybrid ANN	Used trajectory based geographic parameter as an extra input to ANN model; using wavelet transformation decomposed original series into a few sub-series with lower variability
	Fu et al. [113]	RM-GM-FFNN	Enhanced FFNN model with RM and GM to assess the possible correlation between different input variables for improving forecast accuracy
	Song et al. [4]	ANF, Distribution functions,	Proposed interval prediction method and ANF to address the uncertainty of PMs according to the pollutant emission distribution.
Three dimensional models	Luo et al. [27]	Models-3/CMAQ	Provided a method of analyzing the change of pollutants’ concentration in the condition of lacking practical pollution data.
	Grell et al. [92]	Fully coupled online chemistry with the WRF model	The accuracy of forecasting of meteorological modules and chemical modules under different conditions of separation and coupling is explored. The result indicate that the ability to predict a slight increase
Other methods	Kurt et al. [26]	Neural networks based on geographic forecasting models	The models which considered the geographic factor performed better than the models which unconsidered.
	Pan et al. [103]	GM Grey relational analysis	Selected 30 indexes of 5 categories, and find mainly impact factors by using grey relational analysis, then used GM (1, 1) model to forecast the concentration of pollutants
